# Zhi Zi Chi decoction (Gardeniae fructus and semen Sojae Praeparatum) attenuates anxious depression via modulating microbiota–gut–brain axis in *corticosterone combined with chronic restraint stress*‐induced mice

**DOI:** 10.1111/cns.14519

**Published:** 2023-10-31

**Authors:** Xuanhe Tian, Guangyan Wang, Fei Teng, Xiaoyan Xue, Jin Pan, Qiancheng Mao, Dongjing Guo, Xiaobin Song, Ke Ma

**Affiliations:** ^1^ Shandong University of Traditional Chinese Medicine Jinan China; ^2^ Department of Pharmacy, Women and Children's Hospital Qingdao University Qingdao China; ^3^ Shandong Co‐Innovation Center of Classic TCM Formula Shandong University of Traditional Chinese Medicine Jinan China

**Keywords:** anxious depression, bioactive compounds, effector substance, microbiota–gut–brain axis, Zhi Zi Chi decoction

## Abstract

**Background:**

The microbiota–gut–brain axis plays a critical role in neuropsychiatric disorders, particularly anxious depression, and attracts more attention gradually. Zhi Zi Chi decoction (ZZCD) consisting of *Gardenia jasminoides J. Ellis* and *Glycine max* (L.) *Merr*, is a classic formula in clinic and widely applied in anxiety and depression treatment. However, the underlying mechanisms of regulating microbiota–gut–brain axis in the treatment of anxious depression by oral administration of ZZCD remain elusive.

**Materials and Methods:**

In this project, we clarified the origin and preparation methods of the *Gardenia jasminoides J. Ellis* and *Glycine max* (L.) *Merr* and examined the chemical ingredients of ZZCD by liquid chromatograph mass spectrometer. Then, corticosterone combined with chronic restraint stress was applied to establish an anxious depression model. After treated with ZZCD standard decoction, based on enzyme‐linked immunosorbent assay (ELISA), 16S rRNA technology, high‐throughput sequencing, quantitative RT‐PCR and fecal microbiota transplantation (FMT), the multiple associations between nucleus accumbens and intestinal flora in anxious depression mice were determined to clarify the mechanism of ZZCD in the treatment of anxiety and depression disorder.

**Results:**

We found various substances with antidepressant and antianxiety properties in ZZCD such as rosiridin and oleanolic acid. ZZCD could alleviate depressive and anxiety behaviors in anxious depression mice via regulating the disturbance of gut microbiota. Meanwhile, the bioactive compounds of ZZCD might directly active on neurodevelopment and neuroimmune‐related genes. Furthermore, the secretion of prolactin and estrogen, and interfering with mitogen‐activated protein kinase (MAPK) and tumor necrosis factor (TNF) signaling pathways were mainly involved in the multi‐target therapeutic effects of ZZCD against anxiety and depression.

**Conclusions:**

These findings suggested that ZZCD exerts antidepressant effects pleiotropically through modulating the microbiota‐gut‐brain.

## Graphical abstract

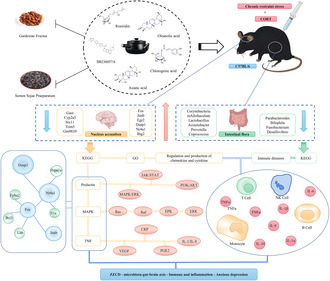

## 1 INTRODUCTION

Anxious depression is the depression accompanied by significant anxiety.^1^ The anxiety symptoms of the disorder are one of the most influential factors contributing to the hindrance of depression treatment.^2^ Patients with anxious depression have more frequent episodes of major depression and are at higher risk for suicidal ideation and suicide attempts than those with non‐anxious depression.^3^ Therefore, finding some safe and effective treatments for anxiety depression will be a top priority.

Depression is closely related to the health status of the brain–gut axis. Ventral tegmental area (VTA) dopamine neurons located in the brain reward circuit play a crucial role in mediating stress responses. Phasic activation of VTA neurons projecting to the nucleus accumbens (NAc), induced susceptibility to social‐defeat stress.^4^ Intestinal flora has become a hot topic for research in treating mental disorders, since they can activate neural pathways and central nervous system (CNS) signaling systems.^5^ Maintaining and restoring the normal state of the intestinal flora is helpful for preventing and treating mental disorders.^6^ Changes in the composition and levels of the intestinal flora result in excessive production of microbial lipopolysaccharide (LPS),^7^ which may indirectly promote hyperfunction of the hypothalamic–pituitary–adrenal (HPA) axis and thus lead to depressive behavior. Therefore, it is reasonable to study the pharmacological treatment for anxiety depression through exploring intestinal flora.^8^

One of the main drugs currently used to treat anxiety depression is venlafaxine.^9^ However, patients often suffer from nausea and vomiting as well as other adverse reactions after taking it.^10^ Venlafaxine can worsen or induce primary sleep disorders, along with extrapyramidal side effects or other side effects such as weight gain and anorexia.^11^ The use of Chinese medicinals for the treatment of psychiatric disorders is increasing to a certain extent in order to avoid the adverse effects by chemical drugs, and the exploration of Chinese medicinal psychopharmacology has received much attention in recent decades.^12^

Zhi Zi Chi decoction (ZZCD), consists of *Gardenia jasminoides J. Ellis* (Also known as gardenia fructus, GF) and *Glycine max* (L.) *Merr* (Also known as semen sojae praeparatum, SSP), both of which have been checked in MPNS (http://mpns.kew.org). It is a traditional Chinese medicine (TCM) formula and exerts anti‐depressive effects by suppressing neuron injury and depression‐like behaviors.^13^ Modern research has shown that the anti‐depression effect of ZZCD might be associated with PKA/CREB/BDNF/TrkB/PSD‐95 pathway influenced by metabolic changes.^14^ In addition, another study also indicated that ZZCD exerts potential antidepressant effects by reversing the imbalance of glutathione and oxidative stress^15^ and increasing short‐chain fatty acid production and anti‐inflammatory bacteria, as well as reducing inflammatory and tryptophan‐metabolizing bacteria.^16^ GF is a Chinese medicinal with a variety of pharmacological functions, such as anti‐inflammatory and antidepressant effect, as well as improving cognition and ischemic brain damage.^17^ SSP is the fermented black soybean. It could be consumed as food besides as a Chinese medicinal.^18^ Studies have shown that SSP can alter depressive‐like behavior in chronically unpredictable mildly stressed rats via the gut microbiota.^19^ A study found that SSP could regulate microbiota, promote butyrate production and activate antioxidant reactions to mitigate gardenia hepatotoxicity when taken with GF.^20^

However, the genuine production area of SSP and GF is not exactly determined, and the mechanism of action of ZZCD is still in the exploration stage. Therefore, exploring the potential targets and pathways of ZZCD for anxiety depression has become the focus of research.

## 2 MATERIALS AND METHODS

### 2.1 Animals

The healthy, specific pathogen‐free (SPF) C57BL/6 mice (male, 5 weeks old) were provided by Beijing Vital River Laboratory Animal Technology Co., Ltd. Mice in each group were fed and watered ad libitum and maintained on a 12 h light/dark cycle with room temperature maintained at (22 ± 2.5) °C and relative humidity of 55 ± 5% and maintained in specific pathogen‐free conditions. All environmental factors such as light conditions, noise levels were strictly controlled as these conditions can greatly affect the stress levels of mice. The experiment followed the National Institutes of Health Guide for the Management and Use of Laboratory Animals and was approved by the Animal Management and Use Committee of Shandong University of Traditional Chinese Medicine with ethical approval number SDUTCM20210301018.

### 2.2 Preparation of ZZCD

GF was purchased from Dengzhou City (Henan Province, China) and black soybeans was bought from Nanyang City (Henan Province, China). Both cities are the genuine producing areas of these two Chinese medicinals, respectively. Both medicinals were verified by Professor J.F. Wang of Shandong University of Traditional Chinese Medicine. The specimens of GF (No. SDUTCM, 20220515‐01) and SSP (No. SDUTCM, 20220515‐02) were stored at Shandong Co‐Innovation Center of Classic TCM Formula, Shandong University of Traditional Chinese Medicine.

Firstly, 1000 g of black soybeans were washed to remove the floating pod skin and immature beans. After that 1200 mL of water were added, then 80 g of *Folium Perillae* and 40 g of *Herba Ephedrae* were added, and the mixture was heated to be boiling at 100°C for 35 min until the soybeans became swelling and soft. Then the soybeans were covered with 100 g of *Folium Perillae* fermented at 37°C for 4 days. When the mixture became hot with white and yellow mold growing all over the surface, it was steamed and then sun‐dried. The final product was SSP.

After the SSP was ready, 17 g of GF was crushed into granules with the diameter ≤8 mm and then decocted with 800 mL of pure water to get 500 mL of decoction. Then 48 g of SSP were added, and the decoction was further decocted to get the final decoction with the volume of 300 mL, which was the standard decoction of ZZCD.

### 2.3 Qualitative analysis of the chemical ingredients of the aqueous decoction of ZZCD based on liquid chromatograph mass spectrometer method

Liquid chromatograph mass spectrometer (LC–MS) analysis for ZZCD, SSP and GF was carried out using a chromatograph (UltiMate 3000 RS, Thermo Fisher Scientific, Shanghai, China) and a mass spectrometer (Q‐Exactive high‐resolution mass spectrometer, Thermo Fisher Scientific, Shanghai, China). C18 column (2.1 mm × 150 mm, 1.8 μm) was used for LC. Mobile phase comprised of water (A) and formic acid (B) (100:0.1). The gradient elution conditions were as follows: 1–20% B in 1–5 min, 20–50% B in 5–10 min, 50–80% B in 10–15 min, 80–95% B in 15–20 min, 95% in 20–27 min, 95–2% B in 27–28 min and 2% in 28–30 min with a flow rate of 0.3 mL/min using 5 μL of sample injection volume. The column oven temperature was kept at 35 °C. Data acquisition and processing were performed using CD2.1 (Thermo Fisher Scientific, Shanghai, China) and the mass analyses were conducted using positive and negative ionizations, respectively.^21^

### 2.4 Establishing anxiety depression model with chronic restraint stress combined with corticosterone and drug intervention

For the stage 1, mice were randomly divided into four groups: Control group, Model group, ZZCD group and Ven group. The animal model of anxiety depression was established by chronic restraint stress (CRS) combined with corticosterone (CORT) injection for 28 days. Restraint stress was administered at 12:00–15:00 daily. CORT suspension (30 mg/kg) was subcutaneously injected at the neck and back after restraint stress was administered. In this project, we followed the principle of using the original volume of the classical TCM prescriptions. According to the original dose reported in *Treatise on Cold Damage and Miscellaneous Diseases*, this dose was converted into the animal dose by ancient/modern dose conversion software for TCM animal experiments which is programmed by our team. After one‐week CORT combined with CRS modeling, the drug intervention was undergone (from the second week to the fourth week) for three consecutive weeks. The ZZCD group was given the ZZCD standard decoction by intragastric administration for three consecutive weeks (12 g/kg/d). Equal volume of saline was administered in the Control and Model groups. The Ven group was given venlafaxine at the dose of 20 mg/kg with the volume of 10 mL/kg.

### 2.5 Behaviors test and digestive tract indexes test

Body weights of mice were measured once a week from the adaptation week for a total of six times. Rectal temperature of the mice was measured every 3 days for a total of 10 times from the first day of the experiment.

The sucrose preference test (SPT) was used to assess the anhedonia of mice. In SPT, a standard housing cage was used with two bottles, one containing sucrose solution (1% (wt/vol)) and one containing regular water. SPT was conducted after 24 h without water or fluids for all mice. During SPT, mice were given 1% sucrose solution and water for 1 h. The sucrose preference ratio was calculated by the following formula: [sucrose intake/(sucrose intake + water intake)] × 100%].

The elevated plus maze test (EPM) and the open field test (OFT) were used to assess anxiety‐like behavior and exploratory activity. In EPM, the mice were placed in the center of the elevated plus maze with the head facing the closed arm. After the mice moved freely for 30 s, the exploration time of the mice entering the open arm within 4 min was recorded. At the end of each mouse experiment, the experimental area was wiped with 75% ethanol.

In OFT, mice were placed alone in an open field of 40 cm × 40 cm × 40 cm and explored freely. To record locomotor activity, a camera was placed above the open box, and mice were allowed 5 min to explore freely. We measured the total distance and time spent in the central area. Before introducing each animal, the test instrument was thoroughly cleaned with 75% ethanol.

The forced swimming test (FST) was used to assess the desperate behavior of mice as described previously. Mice were placed in a plexiglass vessel with a diameter of 10 cm and a height of 25 cm filled with water (23–26°C) at a depth of 10 cm. A camera recorded the 6 min swimming session and immobility of mice was measured in the last 4 min. In this test, immobility was defined as their passive floating in the water.

After behaviors test feces were collected for 16S rRNA sequencing and fecal microbiota transplantation (FMT) before the mice were given nutritive semi‐solid paste containing activated carbon. The digestive system function indexes of CORT combined with CRS‐induced depression model were analyzed by measuring the gastric emptying rates (GER) and small intestinal transit rates (ITR).^22^, ^23^ We comprehensively compared the effects of ZZCD and Verb on the depression and anxiety‐like performance, cognitive‐behavior, and gastrointestinal functions of the mice. All behaviors test were conducted in a soundproofed environment. [Open field (model 63,008), elevated plus maze (model 63,010), strong swimming (model 63,022), SMART3.0 animal behavior video analysis system were purchased from Shenzhen Ruide Technology Co., Ltd].

### 2.6 Measurement of neurotransmitters, pro‐inflammatory cytokines, and HPA axis hormones levels

Then blood samples were taken from the mice orbit for enzyme‐linked immunosorbent assay (ELISA). The expression levels in peripheral blood of three neurotransmitters: 5‐hydroxytryptamine (5‐HT), dopamine (DA) and γ‐aminobutyric acid (GABA), three HPA axis hormone: cortisol (CORT), corticotropin‐releasing hormone (CRH), adrenocorticotropic hormone (ACTH), three pro‐inflammatory cytokines: interleukin (IL)‐6, IL‐1β, and tumor necrosis factor‐α (TNF‐α), and one anti‐inflammatory cytokine IL‐10 were measured using ELISA^24^ (All kits were purchased from Beyotime Biotechnology Co., Ltd., Shanghai, China).

### 2.7 RNA sequencing (RNA‐seq) and bioinformatics analysis

After mice were sacrificed, brain samples were quickly frozen in liquid nitrogen, then placed on ice to separate the NAc tissue and stored in a centrifuge tube at −80°C for RNA‐seq. High‐throughput sequencing of transcriptional profiles was performed by Shenzhen Huada Gene Technology Co., Ltd. And samples with total RNA amount > 10 μg, concentration > 200 ng/μL, RIN ≥8, 28S/18S ratio ≥1.5 were selected for transcriptome construction. The raw sequence data obtained from sequencing were processed with quality control to obtain clean data. DEGs were screened by comparing the model group with the control group and comparing the ZZCD group with the model group, with the following criteria: FPKM > 0.1, fold change ≥ 1.5 or ≤0.66, *p* < 0.05 (3–4 mice per group).

The Gene Ontology (GO) and Kyoto Encyclopedia of Genes and Genomes (KEGG) pathway enrichment analysis of common target proteins were performed using the Metascape database and the R language. And the species was set as “HOMO sapiens”, and the screening condition was set as *p* ≤ 0.05. Visual processing was performed according to enrichment scores.

The Search Tool for the Retrieval of Interacting Genes/Proteins (STRING) database was used to import the targets of ZZCD for anxious depression treatment. The species were set as “Mus musculus” and the confidence was set as “medium confidence (0.400)” to obtain the targets interaction network, which was saved in TSV format and imported into Cytoscape 3.7.0 to construct protein–protein interaction (PPI) network. Topology analysis was performed using the CytoCNA plug‐in. The top six targets of ZZCD for anxious depression treatment were obtained based on the degree of mid‐node connection as the screening condition.

Chemical ingredients of each medicinal from ZZCD were download from PubChem database. Hydrogenation of small molecules and charge calculation was performed using Autodock 4.2.6, and core target structure was obtained using Alpha Fold Protein Structure Database. The obtained structures were import into Autodock. Appropriate grid box for molecular docking was set, and finally the conformation with the lowest docking binding energy was the final docking result, and was visualized with PyMOL 1.8.6. When the docking binding energy is less than 5 kcal/mol, the receptor and ligand can combine spontaneously. The lower the binding energy, the greater the possibility of their combination, the more stable the binding conformation and the greater the possibility of reaction.

### 2.8 Quantitative RT‐PCR

Quantitative reverse‐transcription PCR (qRT‐PCR) was performed to quantify differentially expressed genes (DEGs) in the NAc. Primer sequences are listed in Table S1. Specific qRT‐PCR procedures with a QuantStudio 7 Flex Real‐Time PCR System (Life Technologies, Gaithersburg, Maryland) applied was reported in our previous study.^25^ All operations were performed according to the manufacturer's instructions. The comparative cycle threshold (CT) was applied to calculate the relative expression of mRNAs. The 2^−ΔΔCq^ method was applied to calculate and quantify the relative expression of mRNAs, and β‐actin (AC004; ABclonal Technology, Wuhan, China) was used as a normalization control. Each sample was prepared in triplicate (six mice per group).

### 2.9 16S rRNA sequencing and bioinformatics analysis

16S rRNA sequencing and analysis were performed by Personalbio (Shanghai, China). The total DNA of cecum microorganisms was extracted and amplified. The V3‐V4 region of bacterial 16S rRNA genes was amplified using the forward primer 338 F and reverse primer 806 R, and was sequenced by PE 250 double‐end sequencing using MiSeq Reagent Kit V3. The bioinformatics of each microbial group at different taxonomic levels were analyzed.^26^

The specific composition of various microbial groups at different taxonomic levels was analyzed using the QIIME2 platform, and then the number of taxonomic units of these samples at the seven taxonomic levels of domain, phylum, class, order, family, genus, and species was determined according to the results of taxonomic annotation of sequence. The alpha diversity within each sample was evaluated by calculating ACE index, Chao1 index, Simpson index and Shannon index, and the sparse curve, and rank abundance curve were drawn to evaluate the reliability of sequencing results. Beta diversity refers to the difference in species composition or the rate of replacement of species along environmental gradients among different communities, which is also called between‐habitat diversity. Multi‐dimensional microbial data can be represented by principal coordinate analysis (PCoA) to evaluate the beta‐diversity of samples between groups. Adonis analysis and permutation test were conducted for diversity in homogeny of groups, and dispersion analysis were used to test the differences between groups. The method of LEfSe analysis (LDA > 2) and random forest analysis were used to pre‐screen the potential differential flora among groups, and the LEfSe evolution branch diagram and LDA value distribution histogram were drawn.

### 2.10 Fecal microbiota transplantation

The feces from Control group and ZZCD group were immediately diluted with sterile PBS (1 fecal pellet/ml) for about 15 min, homogenized and then centrifuged at 1000 rpm for 5 min at 4°C to pellet the particulate matter. The final bacterial suspension was mixed with an equal volume of 40% sterile glycerol to a final concentration of 20% under sterile conditions, then stored at 80°C until FMT.

For the stage 2, 18 mice were randomly divided into three groups: Control + Con‐FMT group, Model + Con‐FMT group, and Model + Zzcd‐FMT group. In the antibiotic‐mediated microbiota‐depletion mouse model, ampicillin, gentamicin, metronidazole, neomycin (all at 0.25 mg/day), and vancomycin (0.125 mg/day) were given to all mice for 14 days to deplete gut microbiota. After a three‐day washout period, the recipient Control + Con‐FMT group and Model + Con‐FMT group mice were given 200 μL of donor supernatant from Control group and Model + Zzcd‐FMT group mice were given 200 μL of donor supernatant from ZZCD group by oral gavage every 2 days for 2 weeks.^27^, ^28^ The behavioral tests were performed for four consecutive days from FMT week 2. Blood samples from the mice orbit was used for ELISA.

### 2.11 Statistical analyses

Statistical comparison was performed using unpaired t‐test when only two groups were compared, or by Tukey's test‐corrected one‐way ANOVA or repeated measures ANOVA when more than two groups were compared. The correlations between the relative abundance of microbiota at the genus level and depression and anxious‐like associated features were assessed using Spearman's correlation test in which calculated and plotted using R package “corrplot” (v4.0.3) by Genes Cloud (https://www.genescloud.cn). All data are presented as the mean ± standard error of mean (SEM) and were completed with SPSS 21.0 software (IBM, Armonk, NY). A *p*‐value < 0.05 indicated significant differences for all the statistical tests.

## 3 RESULTS

### 3.1 Preparation of ZZCD

**FIGURE 1:**
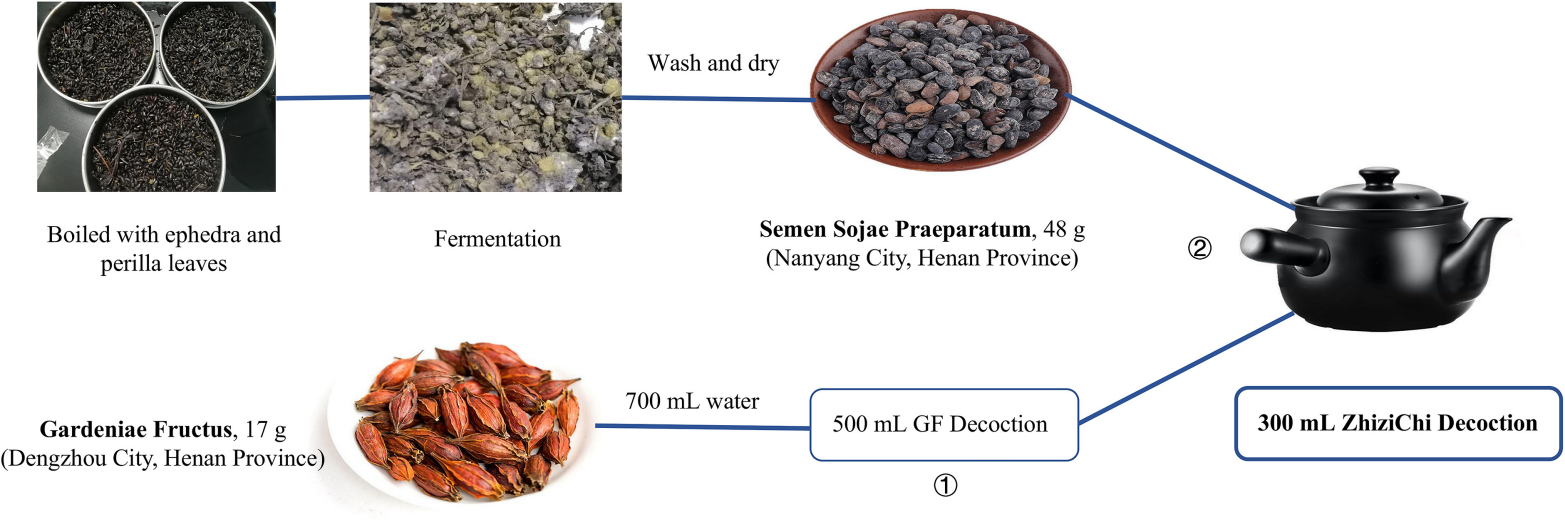
ZZCD standard decoction preparation. The GF is from Dengzhou City (Henan Province, China) and black soybeans from Nanyang City (Henan Province, China). The black soybeans were processed to make SSP. Firstly, 17 g of GF with 800 mL of pure water were decocted to get 500 mL of decoction, and then 48 g of SSP were added in the decoction and further decocted to get 300 mL of decoction.

In order to determine the origin and decoction method of GF and SSP, we reviewed the literature from the Han Dynasty to the present and came to the conclusion that the best producing areas of GF and SSP are Dengzhou City and Nanyang City, respectively, both in Henan Province, China. ZZCD is prepared by decocting 17 g of GF and 48 g of SSP with 800 mL of pure water (Figure 1). Also, we found a traditional preparation method for SSP, which unfortunately is no longer widely used today. According to *Chinese Pharmacopeia*, *Herba Artemisiae Annuae* and *Folium Mori* should be used as auxiliary to make SSP, while in the method found by us *Herba Ephedrae* and *Folium Perillae* should be used as auxiliary in the fermentation process of black soybeans, which might cause the SSP different in the nature and function. SSP prepared by the method found by us is more consistent with the disease mechanism of anxiety depression in TCM, that is, qi stagnation and excessive fire.

### 3.2 Ingredient characteristics of ZZCD

**FIGURE 2:**
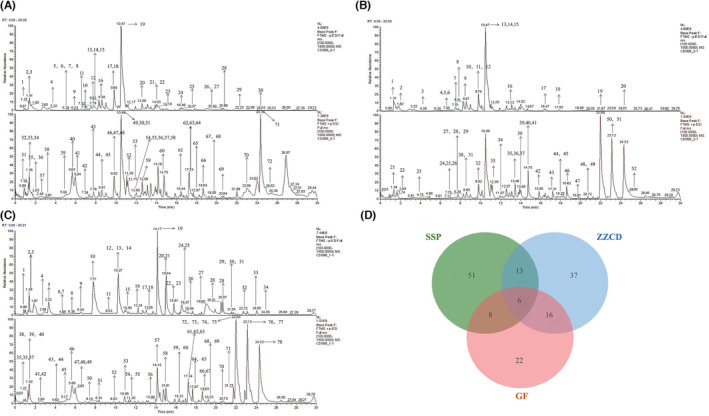
Total ion chromatogram of ZZCD in positive and negative ion mode using UHPLC‐Q‐TOF/MS. (A) Chromatogram of GF on negative and positive ionization mode. (B) Chromatogram of SSP on negative and positive ionization mode. (C) Chromatogram of ZZCD on negative and positive ionization mode. (D) The Venn diagram among three groups. Chromatographic column: AQ‐C18, 150 × 2.1 mm, 1.8 μm, Welch; Flow rate: 0.30 mL/min; Aqueous phase: 0.1% formic acid/water solution; Organic phase: methanol; Washing solution: methanol; Column temperature: 35°C; Autosampler temperature: 10.0°C; Autosampler injection volume: 5.00 μL.

We analyzed the composition of ZZCD, GF, and SSP by high‐performance liquid chromatography combined with mass spectrometry (Figure 2) The results showed that a total of 72 active compounds were detected in ZZCD (Figure 2A and Table 1), 52 compounds were detected in the aqueous decoction of GF (Figure 2B and Table S2), and 78 compounds in the aqueous decoction of SSP (Figure 2C and Table S3). We found that in the decoction 37 new substances were generated (Figure 2D) which are closely related to the target pathways of depression, but have not been developed for medical application, such as (2E)‐4‐Hydroxy‐3,7‐dimethyl‐2,6‐octadien‐1‐ylbeta‐D‐glucopyranoside (rosiridin), asiatic acid, oleanolic acid, and SB236057A.

**TABLE 1:** Characterization of the chemical constituents in ZZCD standard decoction by LC–MS.

Number	Chemical ingredients	Model	RT [min]	Formula	m/z
1	L‐Glutamic acid	−	1.295	C5 H7 N O3	148.06017
2	Acetyl‐L‐carnitine	−	1.419	C15 H10 O5	204.12286
3	Adenine	−	1.427	C16 H18 O9	136.06172
4	Uridine monophosphate (UMP)	−	3.228	C6 H9 N3 O2	323.02872
5	Adenosine 3′5′‐cyclic monophosphate	−	5.235	C25 H24 O12	330.05939
6	6‐Hydroxynicotinic acid	−	5.264	C10 H15 N O	140.03418
7	Gallic acid	−	5.314	C10 H15 N O	169.01332
8	L‐Phenylalanine	−	5.353	C7 H6 O5	166.08627
9	L‐Phenylalanine	−	5.382	C18 H34 O4	164.07072
10	Methylsuccinic acid	−	6.289	C7 H10 O5	131.03375
11	D‐(+)‐Tryptophan	−	7.334	C24 H38 O4	203.08186
12	2‐[3‐(tert‐butyl)‐1‐(4‐fluorobenzyl)‐1H‐pyrazol‐5‐yl]‐5‐(2‐thienyl)‐1,3,4‐oxadiazole	−	7.582	C15 H10 O7	383.13062
13	Methyl 1‐(hexopyranosyloxy)‐5‐hydroxy‐7‐(hydroxymethyl)‐1,4a,5,7a‐tetrahydrocyclopenta[c]pyran‐4‐carboxylate	−	7.895	C7 H12 O6	449.12988
14	(9R,10R)‐10‐(acetyloxy)‐8,8‐dimethyl‐2‐oxo‐2H,8H,9H,10H‐pyrano[2,3‐h]chromen‐9‐yl 2‐methylbutanoate	−	8.29	C22 H36 O12	427.12024
15	Methyl 1‐(hexopyranosyloxy)‐7‐hydroxy‐7‐(hydroxymethyl)‐1,4a,7,7a‐tetrahydrocyclopenta[c]pyran‐4‐carboxylate	−	8.295	C13 H22 N6 O2	449.12949
16	Neochlorogenic acid	−	8.302	C10 H10 O3	353.08755
17	Perillic acid	−	8.589	C21 H34 O11	165.09084
18	Shanzhiside methyl ester	−	9.802	C18 H25 N O	389.14322
19	Ferulic acid	−	9.803	C9 H10 O	227.09088
20	Catechin	−	10.38	C21 H42 O4	289.07193
21	(2E)‐4‐Hydroxy‐3,7‐dimethyl‐2,6‐octadien‐1‐yl beta‐D‐glucopyranoside	−	12.593	C16 H24 O10	377.18152
22	6‐(2‐hydroxy‐3‐methyl‐3‐{[(2S,3R,4S,5S,6R)‐3,4,5‐trihydroxy‐6‐(hydroxymethyl)oxan‐2‐yl]oxy} butyl)‐5,7‐dimethoxy‐2H‐chromen‐2‐one	−	13.909	C6 H14 N4 O2	493.16739
23	6‐O‐Pentopyranosyl‐1‐O‐[(2,6,6‐trimethyl‐1‐cyclohexen‐1‐yl)carbonyl]‐β‐D‐glucopyranose	−	13.989	C10 H14 N2 O5	507.20792
24	N‐{[(2R,3S,4R,5S)‐3,4‐Dihydroxy‐5‐{2‐oxo‐2‐[4‐(2‐pyridinyl)‐1‐piperazinyl]ethyl}tetrahydro‐2‐furanyl]methyl}‐3‐fluorobenzamide	−	14.259	C19 H24 N2 O S	493.17484
25	Estrone	−	16.451	C9 H10 O4	293.15283
26	(3beta, 8xi, 9xi, 14xi, 17xi)‐3‐{[Hexopyranosyl‐(1–6) hexopyranosyl‐(1–4)‐2,6‐dideoxy‐3‐O‐methylhexopyranosyl] oxy}‐14‐hydroxycard‐20(22)‐enolide	−	16.567	C18 H23 N O3	841.42249
27	(3β,5ξ,9ξ,18ξ)‐28‐Hydroxy‐28‐oxoolean‐12‐en‐3‐yl 6‐deoxy‐α‐L‐mannopyranosyl‐(1‐ > 3)‐[β‐D‐glucopyranosyl‐(1‐ > 2)]‐β‐D‐glucopyranosiduronic acid	−	20.455	C9 H20 N2 O2	939.49609
28	9(Z),11(E),13(E)‐Octadecatrienoic Acid methyl ester	−	20.517	C22 H22 F3 N O3	293.24683
29	4‐Methoxy‐butyryl fentanyl	−	20.813	C20 H41 N O2	381.26016
30	Oleanolic acid	−	22.233	C9 H6 O2	439.35608
31	Stearic acid	−	24.058	C5 H12 O5	283.26419
32	D‐(−)‐Aspartic acid	+	1.291	C18 H30 O2	134.04469
33	L‐2‐Aminoadipic acid	+	1.381	C15 H10 O5	162.07594
34	Betaine	+	1.388	C16 H32 O3	118.08636
35	D‐(+)‐Proline	+	1.392	C9 H8 O3	116.07083
36	Leucylproline	+	1.627	C17 H27 N3 O17 P2	229.15453
37	N‐Acetylaspartic acid	+	1.627	C17 H32 O2	176.05528
38	L‐Isoleucine	+	2.487	C16 H12 O5	132.1019
39	Mesaconic acid	+	2.818	C4 H7 N O4	111.00739
40	2‐AI	+	5.06	C13 H24 N2 O	134.09644
41	Pseudoephedrine	+	5.64	C18 H30 O2	148.11182
42	Adenosine 5′‐monophosphate	+	5.873	C21 H18 O12	348.06982
43	Pseudoephedrine	+	5.943	C16 H18 O9	148.11185
44	trans‐3‐Indoleacrylic acid	+	7.337	C5 H5 N5 O	188.07047
45	Geniposidic acid	+	7.882	C19 H32 O2	373.11371
46	2,3,4,9‐Tetrahydro‐1H‐β‐carboline‐3‐carboxylic acid	+	8.647	C21 H32 O11	217.09698
47	7,8‐Dihydroxy‐4‐methylcoumarin	+	8.676	C12 H11 N O3	225.07544
48	Genipin	+	9.815	C11 H9 N O2	207.06535
49	4‐hydroxy‐5,8‐dimethylquinoline‐3‐carboxylic acid	+	9.886	C10 H10 O4	218.08083
50	Chlorogenic acid	+	9.892	C6 H11 N O4	355.1015
51	4‐Hydroxybenzylalcohol	+	10.497	C11 H20 N2 O3	123.04367
52	Geniposide	+	10.503	C10 H14 O2	433.13409
53	4‐Ethylbenzaldehyde	+	10.507	C17 H26 O11	135.08026
54	1,2,3,4‐Tetramethyl‐1,3‐cyclopentadiene	+	11.059	C5 H4 N4 O2	123.11696
55	trans‐Anethole	+	11.11	C10 H8 O4	181.12209
56	Cyclo(phenylalanyl‐prolyl)	+	11.975	C13 H8 N2 O2	245.12827
57	Sinapinic acid	+	11.993	C11 H12 N2 O2	207.06497
58	(2E)‐4‐Hydroxy‐3,7‐dimethyl‐2,6‐octadien‐1‐yl beta‐D‐glucopyranoside	+	12.02	C48 H76 O19	377.18167
59	(2E)‐3‐(2‐{[(2S,3R,4S,5S,6R)‐3,4,5‐trihydroxy‐6‐(hydroxymethyl)oxan‐2‐yl]oxy}phenyl)prop‐2‐enoic acid	+	12.17	C9 H11 N O2	309.09628
60	1‐[4,5‐Dihydroxy‐6‐(hydroxymethyl)‐3‐[(E)‐3‐(4‐hydroxyphenyl)prop‐2‐enoyl]oxyoxan‐2‐yl]oxy‐7‐hydroxy‐7‐methyl‐4a,5,6,7a‐tetrahydro‐1H‐cyclopenta[c]pyran‐4‐carboxylic acid	+	12.173	C6 H9 N O5	521.1665
61	Apigetrin	+	12.244	C9 H11 N O2	433.11227
62	5,7‐Dihydroxy‐2‐(4‐hydroxyphenyl)‐4‐oxo‐4H‐chromen‐3‐yl 6‐deoxy‐alpha‐L‐mannopyranosyl‐(1–2)‐[6‐deoxy‐alpha‐L‐mannopyranosyl‐(1–6)]hexopyranoside	+	12.359	C10 H18 O4	739.20953
63	3‐tert‐Butyladipic acid	+	14.727	C27 H40 N2 O6	201.11253
64	2‐(3‐{[3‐(Tetrahydro‐2H‐pyran‐4‐ylamino)‐3‐oxetanyl]methyl}‐1,2‐oxazol‐5‐yl)ethanol	+	16.449	C16 H24 O8	283.16849
65	Bis(4‐ethylbenzylidene)sorbitol	+	17.244	C9 H8 O4	415.20999
66	2,4‐Dimethylbenzaldehyde	+	17.244	C21 H20 F3 N3 O2 S2	135.08009
67	Cuminaldehyde	+	17.244	C18 H16 O8	149.09572
68	Asiatic acid	+	17.752	C15 H12 O5	487.34305
69	Diisobutylphthalate	+	18.641	C16 H22 O9	301.14026
70	α‐Linolenic acid	+	19.507	C18 H18 N6 O4 S	279.23129
71	(+/−)9,10‐dihydroxy‐12Z‐octadecenoic acid	+	19.526	C6 H6 O6	295.22766
72	(2R)‐1‐(5‐hydroxy‐3‐methylpentyl)‐2,5,5,8a‐tetramethyl‐decahydronaphthalen‐2‐ol	+	20.816	C16 H30 O2	310.30978
73	Oleic acid	+	22.978	C14 H12 F3 N3 O3	281.24857
74	Dodecamethylcyclohexasiloxane	+	24.529	C6 H11 N O3	445.11908
75	SB236057A	+	25.092	C15 H23 N5 O14 P2	535.26947

### 3.3 Effects of ZZCD on the anxiety and depression‐like behaviors of mice

**FIGURE 3:**
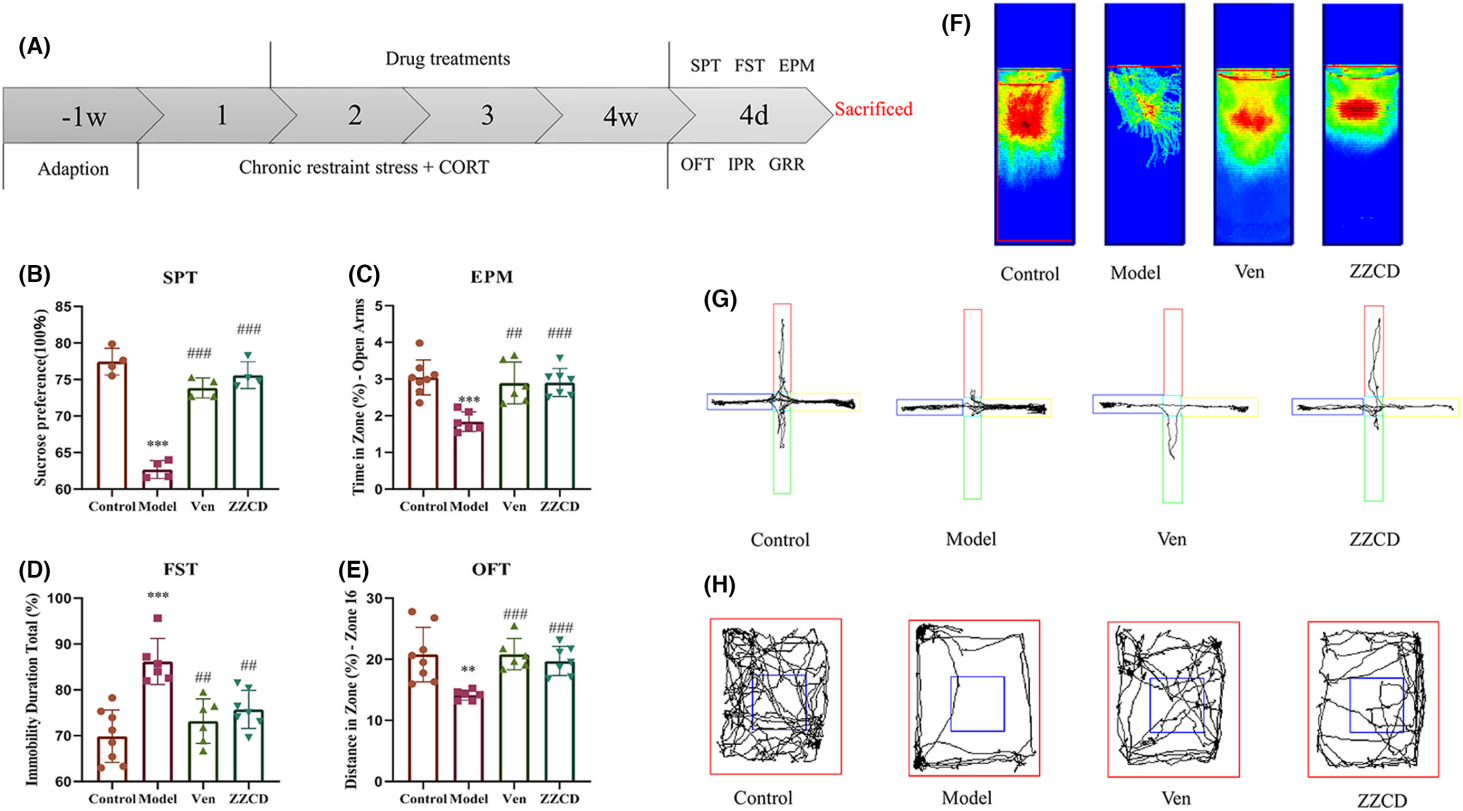
ZZCD attenuates the depressive and anxiety symptoms in mice with anxiety depression. (A) The experimental timeline; (B) Effects of ZZCD on SPT (%) [*F* (2, 9) = 87.77, *p* < 0.0001], (C) Effects of ZZCD on EPM (%) [*F* (2, 16) = 13.11, *p* = 0.0004]; (D) Effects of ZZCD on OFT (%) [*F* (2, 16) = 17.96, *p* < 0.0001]; (E) Effects of ZZCD on FST (%) [*F* (2, 16) = 13.24, *p* = 0.0004]; (F) Comparison of heat map with struggle trajectory in FST; (G) Comparison of movement trajectory in EPM with the vertical grid on the open arm. (H) Comparison of movement trajectory in OFT. Data are expressed as mean ± standard error of mean (SEM) (*n* = 6–8 per group) and differences were compared by One‐Way ANOVA. **p < 0.01 and ***p < 0.001 versus the Control group; ^##^ *p* < 0.01 and ^###^ *p* < 0.001 versus the Model group. Model: CORT combined with chronic restraint stress (CRS) + Saline, Ven: CORT combined with CRS + Ven treatment, ZZCD: CORT combined with CRS + ZZCD treatment.

We firstly compared effects of GF, SSP and three different doses of ZZCD, which equivalent to the amount of high (18 g/kg/d), medium (12 g/kg/d), and low (6 g/kg/d) concentration in human body. Behavioral tests showed the original dose of anti‐anxiety and depression effect is better than GF, SSP and other two concentrations of ZZCD (Figure S1). Of note, optimal drug was selected to effect concentration to carry following mechanism associated experiments. CORT combined with CRS could decrease body weights and increase rectal temperature of mice (all *p* < 0.001, Figure S2 and Table S4). We found that ZZCD interfered with anhedonia, social behavior, and despair behavior of mice in various behaviors tests (Figure 3). In the SPT, ZZCD significantly modulated the preference for sugar water in anxiously depressed mice (*p* < 0.001), and increased the struggle time in FST to reduce despair behavior (*p* < 0.01); in the social behavior evaluation test, EPM and OFT, ZZCD also improved anxious behavior (*p* < 0.001), the total speed in EPM and OFT of ZZCD and Ven groups were faster than Model group (all *p* < 0.001, Figure S3). The total distance and the time spent in the center zone of OFT, total distance, and the distance in zone‐open arms of EPM are shown in Table S5.

### 3.4 Effects of ZZCD on the neurotransmitters, pro‐inflammatory cytokines, and HPA axis hormones levels of peripheral blood

**FIGURE 4:**
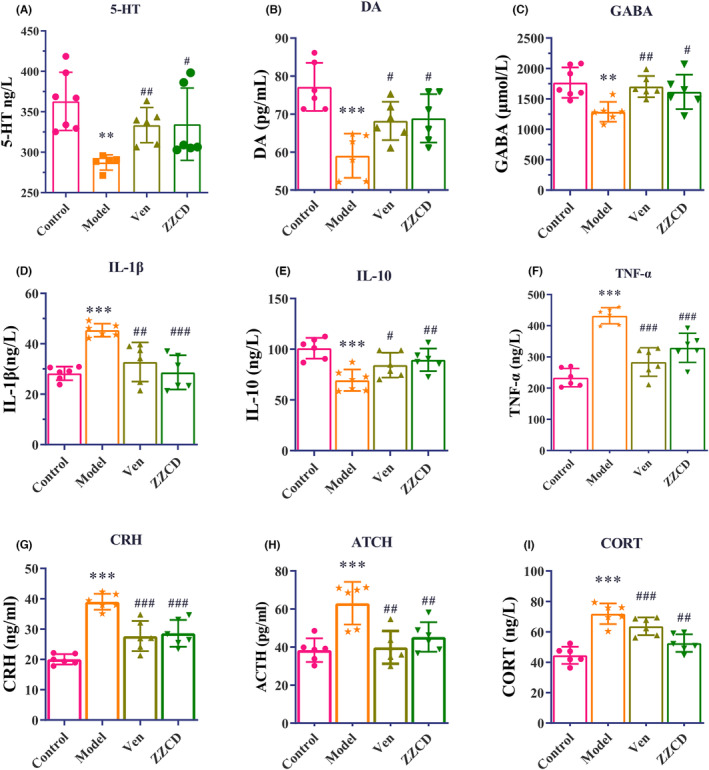
ZZCD attenuates levels of neurotransmitters, pro‐inflammatory cytokines and HPA axis hormones in the peripheral blood of mice with anxiety depression. ZZCD significantly increased the levels of (A) 5‐HT (ng/L) [*F* (2, 14) = 4.242, *p* = 0.0363], (B) DA (pg/L) [*F* (2, 15) = 5.467, *p* = 0.0165], (C) GABA (μmol/L) [*F* (2, 15) = 6.231, *p* = 0.0107], (D) IL‐1β (ng/L) [*F* (2, 15) = 12.11, *p* = 0.0007], (E) IL‐10 (μg/L) [*F* (2, 16) = 5.611, *p* = 0.0142], (F) TNF‐α (ng/L) [*F* (2, 15) = 6.231, *p* = 0.0107], (G) CRH (ng/mL) [*F* (2, 15) = 13.86, *p* = 0.0004], (H) ATCH (pg/mL) [*F* (2, 15) = 10.21, *p* = 0.0016], and (I) CORT (ng/L) [*F* (2, 15) = 14.85, *p* = 0.0003]. Data are expressed as mean ± standard error of mean (SEM) (*n* = 6 per group) and differences were compared by One‐Way ANOVA. **p < 0.01 and ***p < 0.001 versus the control group; ^##^ *p* < 0.01 and ^###^ *p* < 0.001 versus the Model group. Model: CORT combined with CRS + Saline, ZZCD: CORT combined with CRS + ZZCD treatment.

We determined levels of 5‐HT, DA, GABA, IL‐1β, IL‐10 TNF‐α, CRH, ACTH, and CORT in the peripheral blood by ELISA to investigate whether ZZCD treatment could alter neurotransmitters, pro‐inflammatory cytokines, and HPA axis hormone in anxious depression mice (Figure 4). CORT combined with CRS significantly reduced levels of three neurotransmitters (5‐HT, DA and GABA) and anti‐inflammatory factor IL‐10 (both *p* < 0.01), increased three HPA axis hormones (CRH, ACTH and CORT), and two proinflammatory factors (IL‐1β and TNF‐α) in peripheral blood (both *p* < 0.001). Ven and ZZCD could significantly reduce levels of these substances. After treated with ZZCD, levels of CRH (*p* < 0.001), ACTH, CORT (both *p* < 0.01), IL‐1β and TNF‐α (both *p* < 0.001) were reduced. ZZCD also increased levels of 5‐HT, DA, GABA (both *p* < 0.05), and IL‐10 (*p* < 0.01).

### 3.5 Effects of ZZCD on mRNA in NAc based on high‐throughput sequencing, molecular docking verification, and quantitative RT‐PCR

**FIGURE 5:**
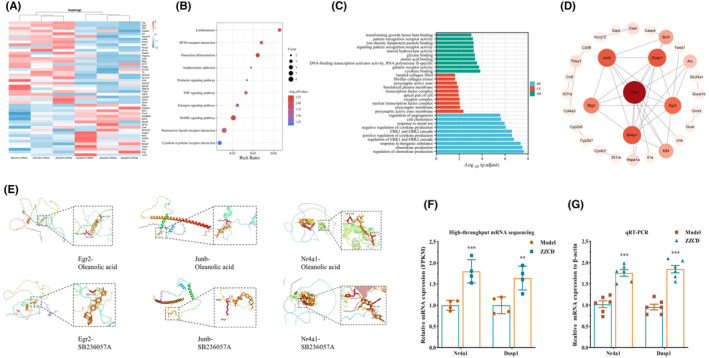
Effects of ZZCD on the NAc in mice with anxiety depression. (A) Heat map of differential genes in NAc of ZZCD and Model groups (*n* = 3 per group). (B) KEGG enrichment analysis map of the potential treatment pathways of ZZCD for anxiety depression. (C) The GO enrichment map. (D) The PPI network map. (E) Molecular docking analyses of the molecular interactions and binding modes of compounds with the active sites of target genes. (F) The relative mRNA expression levels of *Nr4a1* and *Dusp1* quantified by high‐throughput mRNA sequencing (*n* = 4 per group). (G) The relative mRNA expression levels of *Nr4a1* and *Dusp1* quantified by qRT‐PCR (*n* = 6 per group). Data are expressed as mean ± SEM, *p < 0.05, **p < 0.01, and ***p < 0.001 versus the Control group; ^#^ *p* < 0.05, ^##^ *p* < 0.01, and ^###^ *p* < 0.001 versus the Model group. Model: CORT combined with CRS+ Saline, ZZCD: CORT combined with CRS + ZZCD treatment.

We used high‐throughput sequencing and quantitative RT‐PCR to investigate the effects of ZZCD on NAc in mice (Figure 5). Using high‐throughput sequencing, we obtained 49 differential genes of the NAc in each group of mice among which mRNAs such as *Fos*, *Junb*, *Egr2*, *Dusp1*, *Nr4a1*, and *Btg2* were upregulated in the ZZCD group, *Gast*, *Cyp2a5*, *Stx11*, and *Trim5* were downregulated in the ZZCD group (Figure 5A and Table S6). The result of KEGG showed the differential genes of NAc were mainly enriched in neuroactive ligand/receptor interaction, prolactin, estrogen, mitogen‐activated protein kinase (MAPK), tumor necrosis factor (TNF) signaling pathway and other pathways (Figure 5B), GO enrichment showed that the differential genes were mainly involved in the positive and negative regulation and binding of cytokine production, amino acid binding and other processes (Figure 5C). Six core mRNAs on which ZZCD works for anxiety depression were obtained by constructing PPI network: *Fos*, *Junb*, *Egr2*, *Dusp1*, *Nr4a1*, and *Btg2* (Figure 5D).

Molecular docking of the screened chemical ingredients from ZZCD with the core mRNAs was conducted. The binding energy of the main active ingredient and the main target was set at less than less than 5 kcal/mol. The lower the binding energy is, the higher the binding activity is, and the easier the compound binds to the target. Our molecular docking results showed that the key gene and metabolite could form a stable conformation. Visualization of the docking results of all molecules was conducted. Among them, the key genes *Egr2*, *Junb* and *Nr4a1* showed the best docking results with oleanolic acid and SB236057A (Figure 5E and Table S4).

The qRT‐ PCR was conducted to verify the results of the high‐throughput sequencing. We analyzed two significantly changed mRNAs (*Nr4a1* and *Dusp1*) of the high‐throughput sequencing between ZZCD and Model group. The mRNA expression levels of *Nr4a1* and *Dusp1* (both *p* < 0.01) significantly increased in mice administered ZZCD (Figure 5F,G). We could support our research hypothesis by the results from qRT‐PCR.

### 3.6 The modification effect ZZCD on digestive system and intestinal flora in anxious depression mice

**FIGURE 6:**
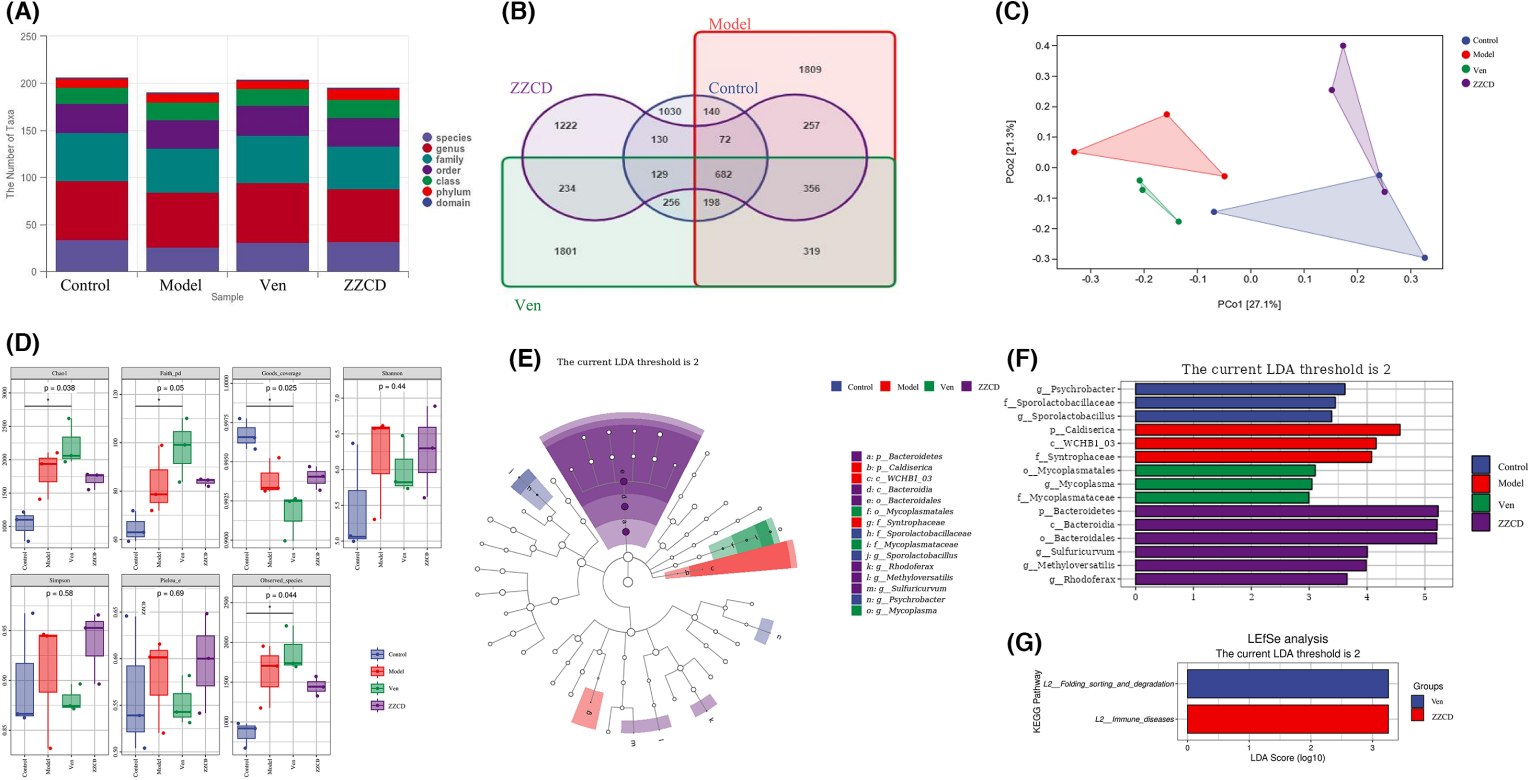
Analysis of gut microbiota following drug treatment. (A) Histogram of the number of OTUs in the flora from samples of cecum contents. (B) Venn diagram of differences in OTU distribution. (C) PCoA analysis. (D) Alpha‐diversity analysis. (E) LEfSe evolutionary branching diagram. Different colors represent different groups. (F) Histogram of LDA value distribution. (G) Predicted targets of flora action at the genus level. (*n* = 3 per group).

**FIGURE 7:**
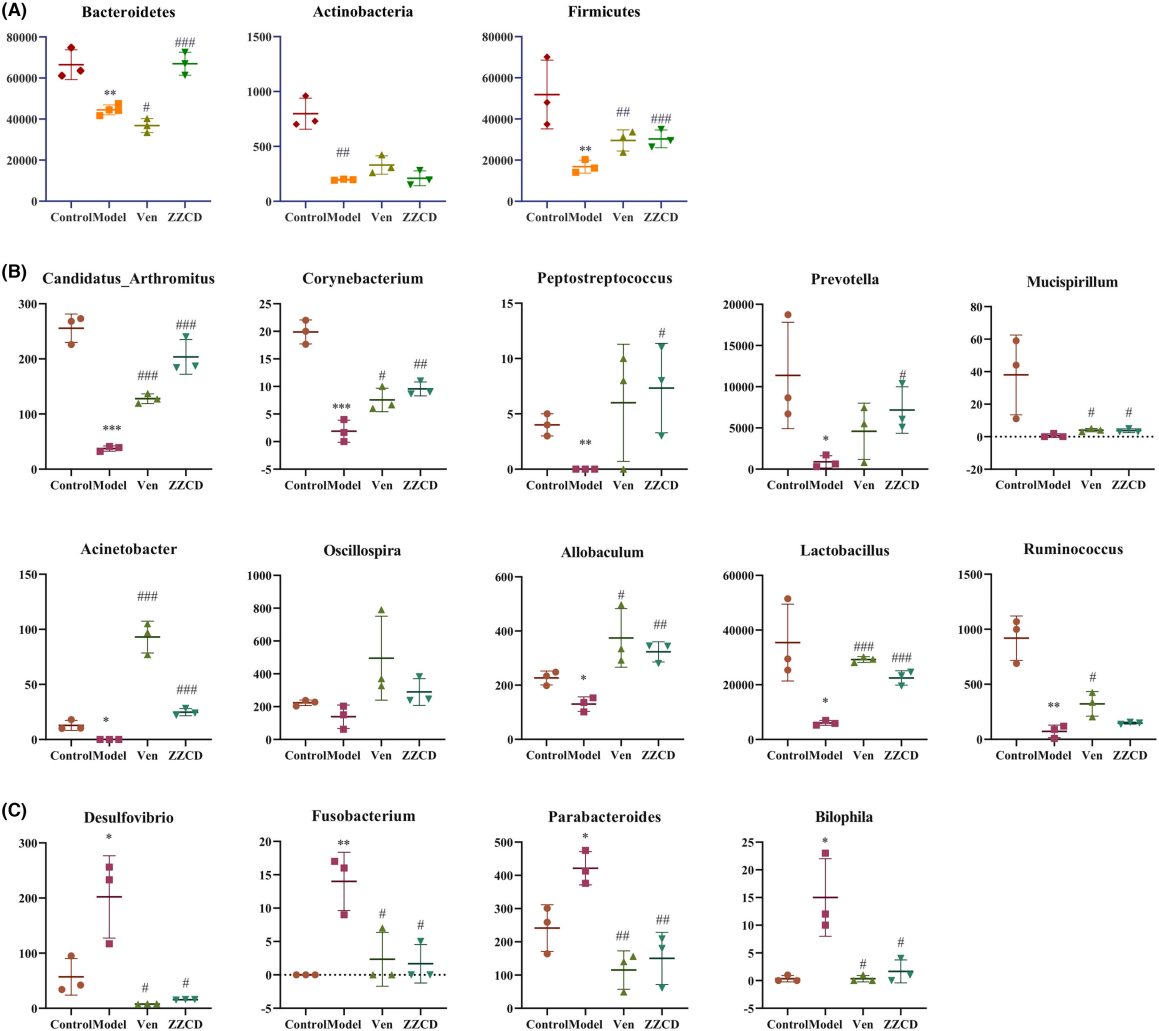
ZZCD can significantly affect the intestinal flora in mice with anxiety depression. (A) Histogram of the differences in the abundance of bacterial populations at the phylum level. (B,C) Histogram of the differences in the abundance of bacterial populations at the genus level. Data are expressed as mean ± SEM (*n* = 3 per group), and differences were compared by One‐Way ANOVA. *p* < 0.05, **p < 0.01, and ***p < 0.001 versus the Control group; ^#^ *p* < 0.05, ^##^ *p* < 0.01, and ^###^ *p* < 0.001 versus the Model group. Model: CORT combined with CRS + Saline, ZZCD: CORT combined with CRS + ZZCD treatment.

**FIGURE 8:**
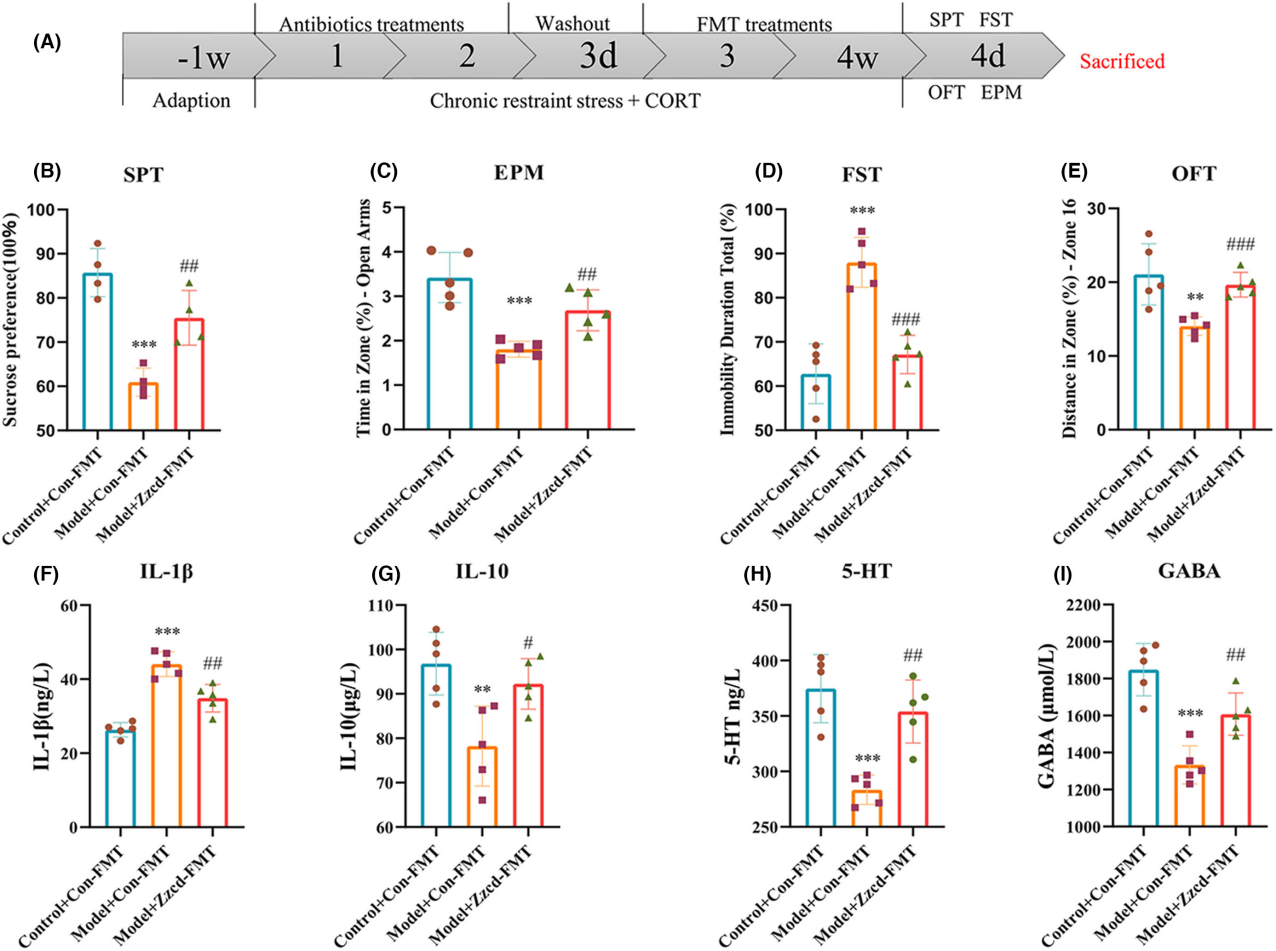
The donor supernatant of ZZCD group can significantly affect depressive and anxiety symptoms in mice with anxiety depression. (A) The experimental timeline; Effects of the donor supernatant of ZZCD group on (B) SPT (%), (C) EPM (%), (D) FST (%), (E) OFT (%), (F) IL‐1β (ng/L), (G) IL‐10 (μg/L), (H) 5‐HT (ng/L), and (I) GABA (μmol/L). Data are expressed as mean ± SEM (*n* = 5 per group) and differences between two groups were compared by *t*‐test. *p < 0.05, **p < 0.01, and ***p < 0.001 versus the Control + Con‐FMT group; ^#^ *p* < 0.05, ^##^ *p* < 0.01, and ^###^ *p* < 0.001 versus the Model + Con‐FMT group. Model + Con‐FMT: CORT combined with CRS + donor supernatant of Control group, Model + Zzcd‐FMT: CORT combined with CRS + donor supernatant of ZZCD group treatment.

We used 16S rRNA sequencing to investigate the effects of ZZCD on intestinal flora in anxious depression mice (Figures 6 and 7) and used FMT to verify it (Figure 8).

In this part, anxiety‐depressed mice showed a significant dysregulation of their intestinal flora. First, we analyzed two digestive system indicators, ITR, and GER (Table S7). The results showed that ITR and GER in ZZCD, venlafaxine and control group were higher than those in model group. The sequences with 97% similarity obtained by sequencing were grouped as one operational taxonomic unit (OTU). The number of OTUs in Control, Model, Ven, and ZZCD groups were 1030, 1809, 1801, and 1222 respectively (Figure S4). The total number of OTUs among the four groups was 682. The total number of OUT in the Model and Control groups was 140; in Ven and ZZCD groups was 256 and 130, respectively, compared with the Control group; in Ven and ZZCD groups was 319 and 257 compared with the Control group; in Ven and ZZCD groups was 234. The total number of OTUs in the Ven, ZZCD, and Model groups was 356, and Model groups was 356. The total number of OTUs among Ven, ZZCD, and Control groups was 129 (Figure 6A,B).

The PCoA analysis method is one of the most classical unconstrained ranking methods. The Bray Curtis algorithm in PCoA analysis was used to estimate the distances between samples, and the principal coordinates with the most significant contribution were selected for combination. The closer the projection distance of the samples on the axes, the more similar the community composition of these two samples in the corresponding dimension. The results of PCoA analysis among the four groups showed that the Control group was separated from the Model group and located on both sides of the two‐dimensional map; ZZCD was the closest distance to the Control group (Figure 6C).

In the alpha diversity analysis, Chao1 index and observed species index were used to assess the community richness of the colony, and the Shannon index and Simpson index were used to calculate the community diversity. The alpha diversity index of the model group increased significantly (*p* < 0.05) (Figure 6D). We constructed rarefaction curves that could reflect the sequencing depth of samples based on the number of extracted sequences and the number of OUTs. The flatness of the curves reflects the effect of sequencing depth on sample diversity. The curves of the four groups of samples became smoother and smoother, indicating the sequencing depth meeting the requirements, and the sequencing results fully reflecting the diversity of the current samples (Figure S5).

The Control, Model, Ven, and ZZCD groups were pre‐screened for LEfSe differential analysis (Figure 6E,F) and random forest analysis at the phylum, family, and genus levels (Figures S6–S8) was conducted. We predicted the targeting pathways for differential flora at the genus level in the ZZCD and Ven groups. We found that venlafaxine could intervene folding sorting and degradation, and the intestinal flora metabolites intervened by ZZCD are associated with immune diseases (Figure 6G).

CRS combined with CORT injection could significantly reduce the abundance of *Bacteroidetes*, *Actinobacteria*, and *Firmicutes* in the intestine of mice at the phylum level (*p* < 0.01), while ZZCD could significantly restore the abundance of *Bacteroidetes* and *Firmicutes* (Figure 7A). At the family level, the abundance of *Mogibacteriaceae*, *Corynebacteriaceae*, *Enterobacteriaceae*, *F16*, and *Turicibacteraceae* were significantly reduced. The abundance of *Bacteroidaceae* was more significantly increased in the Model than Control group, while ZZCD had a significant effect on the abundance of *Bacteroidaceae* compared with Model group (Figure S9). At the genus level, ZZCD significantly increased the abundance of *Candidatus*, *Arthromitus*, *Corynebacterium*, *Allobaculum*, *Lactobacillus*, *Acinetobacter*, *Peptostreptococcus*, and *Prevotella* (Figure 7B), and decreased the abundance of *Parabacteroides*, *Bilophila*, *Fusobacterium*, and *Desulfovibrio* (Figure 7C).

FMT was conducted to verify that ZZCD could improve anxiety and depression in mice through gut microbiota (Figure 8). After CORT combining with CRS and treating with donor supernatant from Control group, mice from Model + Con‐FMT group showed a significant increase in anxiety and depressive behaviors. And the levels of neurotransmitters (5‐HT and GABA) (both *p* < 0.001) and IL‐10 (*p* < 0.01) in peripheral blood were decreased, the level of IL‐1β was increased (*p* < 0.001). In the group of treating with donor supernatant from ZZCD group, the depressive behaviors were decreased in SPT (*p* < 0.01) and FST (*p* < 0.001), anxiety behaviors were decreased in EPM (*p* < 0.01) and OFT (*p* < 0.001), and the levels of 5‐HT, GABA (both *p* < 0.01) and IL‐10 (*p* < 0.05) were increased and the level of IL‐1β (*p* < 0.01) was decreased.

### 3.7 Depression and anxious‐like behaviors, digestive system indicators, neurotransmitters, inflammatory factors, and targets of the mice treated with ZZCD correlate with gut microbes

**FIGURE 9:**
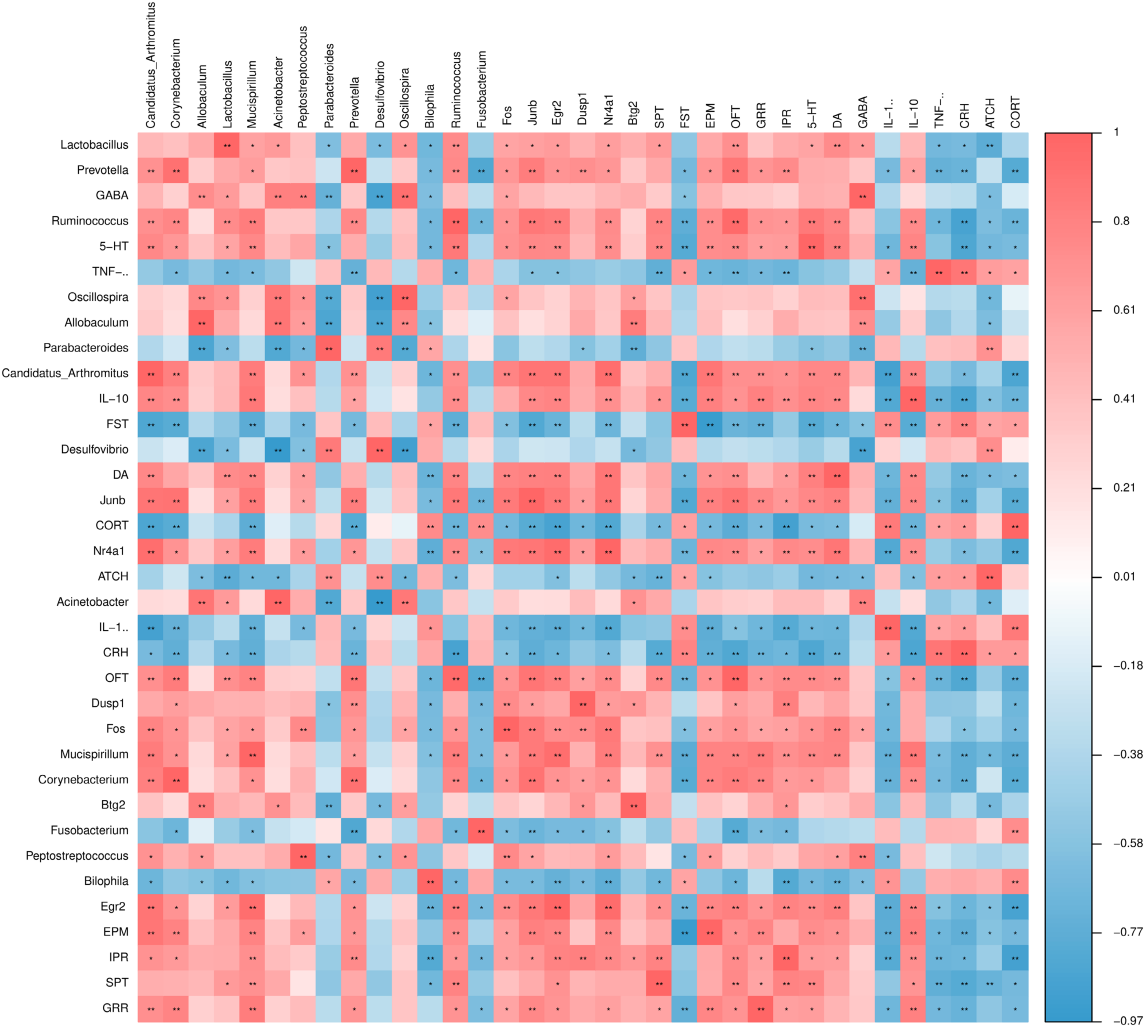
Spearman correlations between gut microbiota at genus level, anxiety and depression‐like behaviors, digestive system indicators, neurotransmitters, inflammatory factors, and core differential mRNAs of mice treated with ZZCD. Spearman's rank correlation coefficient among 2 digestive system indicators, 6 core differential mRNAs, 5 depression‐like behaviors, 3 neurotransmitters, 3 inflammatory factors, 3 HPA axis hormones and 14 gut microbiota that differed significantly in abundance between different treatment groups. Results of correlation and significance test, *p < 0.05, **p < 0.01, ***p < 0.001. Model: CORT combined with CRS + Saline, ZZCD: CORT combined with CRS + ZZCD.

We analyzed the associations between the six core mRNAs, genus‐level intestinal flora, two digestive system indicators, three neurotransmitters, three inflammatory factors, three HPA axis hormones, and four behavioral indicators (Figure 9). The results showed that the percentage of immobility time in FST mice in behavior, and *Parabacteroides*, *Desulfovibri*, *Bilophila*, and *Fusobacterium* in flora negatively correlated with the remaining indicators trend. According to these results, we concluded that changes in the gut microbiota were associated with anxiety and depression‐like behaviors, digestive system indicators and mRNAs.

## 4 DISCUSSION

We attempted to elucidate the mechanism of ZZCD in treating anxious depression from the microbiota–gut–brain axis perspective by constructing multiple associations between NAc‐intestinal flora in anxious depression mice. Firstly, we determined the chemical ingredients of ZZCD, SSP, and GF. We found that when decocted with GF SSP could produce new compounds with anti‐inflammatory, sedative, and antidepressant effects, such as oleanolic acid and SB236057A. Oleanolic acid could inhibit edema and was of significant inhibitory activity against inflammation by activating the pituitary‐adrenocortical system, inhibiting the synthesis or release of prostaglandins (PGs), suppressing endothelial endotoxin‐mediated high‐mobility group box protein 1 (HMGB1) release or regulating MAPK, PI3K/Akt/NF‐κB/ICAM‐1/JAK/STAT signaling pathways.^29^ Inflammation and immune regulation are potential drug targets for the treatment of depression. Oleanolic acid may achieve the treatment of depression by exerting anti‐inflammatory effects.^30^ Oleanolic acid produced antidepressant‐like effects in mice exposed to chronic stress, while decreased SGK1 and activated BDNF‐AKT/mTOR signaling in the hippocampus in an animal model of CORT‐induced depression.^31^ SB236057A is a potent selective 5‐HT1B receptor inverse agonist that increases extracellular 5‐HT levels in the guinea pig dentate gyrus without significant side effects.^32^ Thus, the acute 5‐HT1B receptor blocker SB236057A could be used as an antidepressant providing a rapid onset of action.^33^

TCM classifies the symptoms similar to anxious depression as stagnated qi transforming into fire. We use ZZCD to treat it according to “dispersing fire for treating depressing of fire” from *Huangdi's Internal Classic*. GF in ZZCD can clear the heat to treat anxiety and SSP can dispel melancholy to treat depression. In the analysis of mice behaviors, the reducing of preference for sugar water of the Model group in SPT represented the absence of pleasure, and the reducing of struggle of the Model group in FST represented the desperate state of the mice. Both of them suggested the depressive state of the mice. Meanwhile, the significantly reducing of the social behavior of the Model group in EPM and OFP suggested the anxious state of the mice. ZZCD was effective in interfering with both the anxiety and depressive behaviors. Therefore, it is suggested that chronic restraint stress combined with corticosterone injection is an effective method for constructing anxiety‐depression models, and ZZCD can significantly reverse the anxiety‐depression state caused by it.

According to results of ELISA, ZZCD can exert antidepressant and anxiety effects by reducing the levels of pro‐inflammatory factors (IL‐1β and TNF‐α) in peripheral blood, increasing the levels of anti‐inflammatory factors (IL‐10) and neurotransmitters (5‐HT, DA and GABA), and regulating HPA axis hormones (CRH, ACTH, and CORT). Similar to our results, previous reports have shown changes in these neurotransmitters, pro‐inflammatory cytokines and HPA axis hormones in the depression mice.^34^, ^35^, ^36^ These results indicate that ZZCD can intervene in depression by regulating the HPA axis and peripheral immunity.

According to KEGG pathway enrichment analysis, differential genes in the NAc showed that the pathways were mainly enriched in extracellular matrix (ECM)‐receptor interaction, osteoclast differentiation, cytokine‐cytokine receptor interaction, and estrogen signaling pathway. In a study using bioinformatics analysis to identify key genes and diagnostic value of major depression, analysis of pathway enrichment analysis similarly revealed that DEGs are mainly involved in hematopoietic cell lineage, PI3K/Akt signaling pathway, and cytokine‐cytokine receptor interaction.^37^ These findings suggested that the regulation of ECM is a key mechanism shared by different gut microbiota and that the inhibition of energy metabolism in hypothalamus by gut microbiota from major depressive disorder (MDD) patients is a potential mechanism for behavioral regulation and depression.^38^ In a study in which transcriptome analysis was conducted to evaluate depression, it was found that osteoclast differentiation was upregulated in patients with bipolar disorder.^39^

Studies have shown that estrogen is highly involved in a wide range of brain functions such as cognition, memory, neurodevelopment and neuroplasticity. The contribution of estrogen receptors and estrogen signaling to cognition and neuroprotection by mediating a variety of neural systems including dopaminergic, serotonergic, and glutamatergic systems is important.^40^ Abnormalities in neuroactive ligand‐receptor interaction signaling pathways can induce neurotoxicity affecting neurotransmitters.^41^ The GO enrichment analysis showed that thought regulating cytokine and chemokine ZZCD intervene anxious depression. Increased levels of anxiety and depressive symptoms may promote changes in specific functional chemokines associated with a chronic inflammatory process.^42^ And the inflammation hypothesis of depression suggests that cytokines play a key role in the pathophysiology of MDD and alterations in peripheral cytokine levels are associated with antidepressant treatment outcome.^43^ Through the PPI network we found that the potential targets of ZZCD for the treatment of anxiety and depression are *Fos*, *Nr4a1*, *Dusp1*, *Junb,* and *Egr2*. It was found that *Fos* and *Dusp1* were the mRNAs for biopsychological stress marker genes associated with major depression.^44^ The transcriptional control pathway of MDD after treatment revealed a significant high expression of the gene *Egr2* in the upregulated promoter.^45^ We determined the core mRNAs and made molecular docking with oleanolic acid and SB236057A.The result showed that the key gene and metabolite can form a stable conformation. Therefore, we speculate that the combination of GF and SSP can exert a multi‐component, multi‐target effect synergistically, and that the two Medicinals decocted together can increase sedative and antidepressant effects mutually.

Since the studies on *Fos* and *c‐Fos* are abundant, we chose to study the expression levels of *Nr4a1* and *Dusp1*. *Nr4a1* is an activity‐dependent transcription factor rapidly transcribed in response to neuronal activity and stress.^46^ *Nr4a1*, a key mediator during the regulation of inflammatory disease factors,^47^ could inactivate the MAPK/ERK/CREB signaling pathway.^48^ Up‐regulation of *Nr4a1* expression may inhibit oxidative stress induced volatile antidepressant effects.^49^ According to clinical studies, the expression level of inflammatory factor *Nr4a1* was significantly downregulated in the transcriptome sequencing of the dentate gyrus in the MDD patients compared with the control, suggesting that neuroinflammation plays a crucial role in MDD.^50^ *Dusp1* is a dual‐specificity phosphatase that belongs to a family of phosphatases that inactivate MAPKs, a family of key regulators of cellular processes and the immune response. *Dusp1* overexpression appears to be connected to chronic stress.^51^ *Dusp1* could promote downstream ERK/BDNF signaling to treat depression^52^ and MAPKs/NF‐κB signaling to decrease the level of TNF‐α.^53^ Overexpression of *Dusp1* is vital to the physiology of stress and linked to a depressive‐like phenotype, which is a common feature of various rodent chronic‐stress‐based models of depression.^54^

In the intestinal flora analysis for the ZZCD anti‐depression and anxiety effect we found a significant increase in alpha index in the Model group, ZZCD group and Ven group suggesting successful establishment of animal models. At the genus level ZZCD increased the abundance of those probiotics like *Corynebacterium*, *Allobaculum*, *Lactobacillus*, *Acinetobacter*, and *Prevotella* for depression. A study showed that *Acinetobacter* and *Lactobacillus* were beneficial bacteria in depressed rats.^55^ *Corynebacterium* decreased in abundance in the flora analysis of depressed mice.^56^ *Allobaculum* is a probiotic with anti‐inflammatory,^13^ hypoglycemic, and hypolipidemic effects.^57^ *Prevotella* has a significant anti‐inflammatory effect and thus promotes the expression of BDNF in the hippocampal nerve to exert antidepressant effects.^58^ ZZCD also reduced the abundance of harmful bacteria like *Parabacteroides*, *Bilophila*, *Fusobacterium*, and *Desulfovibrio*. The increased abundance of *Parabacteroides* induced depressive‐like behavior in mice.^59^ In a study it was found that the abundance of *Bilophila* in the feces of patients with major depression was 2.5 times higher than that of the healthy population.^60^ *Desulfovibri* was significantly enriched in the feces of chronic unpredictable mild stress (CUMS)‐treated depressed mice.^61^ Fecal microbiota transplantation is a microbiota‐targeted technique. The donor's gut microbiota could be infusion into the recipient, changing the gut microbial community significantly to treat depression.^62^ After treated with donor supernatant from ZZCD group, anxiety, and depression behaviors of mice were improved. The levels of neurotransmitters (5‐HT and GABA) and anti‐inflammatory factor (IL‐10) in peripheral blood were increased, the level of pro‐inflammatory factor (IL‐1β) was decreased. The results of FMT verified that ZZCD could improve anxiety and depression in mice through gut microbiota.

We analyzed the associations between the six core mRNAs, 14 genus‐level intestinal flora, two digestive system indicators, three neurotransmitters, three inflammatory factors, three HPA axis hormones, and six behavioral indicators by spearman correlation analysis to construct the associations between gut and brain. The result of spearman corroborates the experimental results and further supports that ZZCD improves depression‐like behaviors through “gut‐brain” regulation. Taken together, ZZCD has a positive effect on the antidepressant and anxiolytic effect by elevating the abundance of probiotics such as *Corynebacterium*, *Allobaculum*, *Lactobacillus*, and reducing the abundance of harmful bacteria such as *Parabacteroides*, *Bilophila*, *Fusobacterium*, *Desulfovibrio*. At the same time, ZZCD could affect and participate in the process of neuroactive ligand/receptor interaction, regulate the HPA axis and secretion of prolactin and estrogen, and interfere with MAPK and TNF signaling pathways to reduce the level of inflammation to treat anxiety depression.

These findings provide an insight into possible mechanism underlying the multi‐component, multi‐target, and multi‐pathway therapeutic effects of ZZCD on anxious depression. ZZCD could be developed as a new, safe, and effective drug for anxious depression treatment potentially. It should be noted, however, that all of the above conclusions are based on the results of this study, and should be further validated. Therefore, more experiments on the antidepressant effect and safety of ZZCD should be conducted.

In the future, the components of this SSP will be qualitatively analyzed and compared with the SSP recorded in the *Chinese pharmacopeia*. And we plan to carry out further studies to find out what is the optimal dosage of the substances in ZZCD to treat anxious depression. We hope that those future works will help to further elucidate the potential of ZZCD as a potential treatment for anxious depression.

## 5 CONCLUSION

**FIGURE 10:**
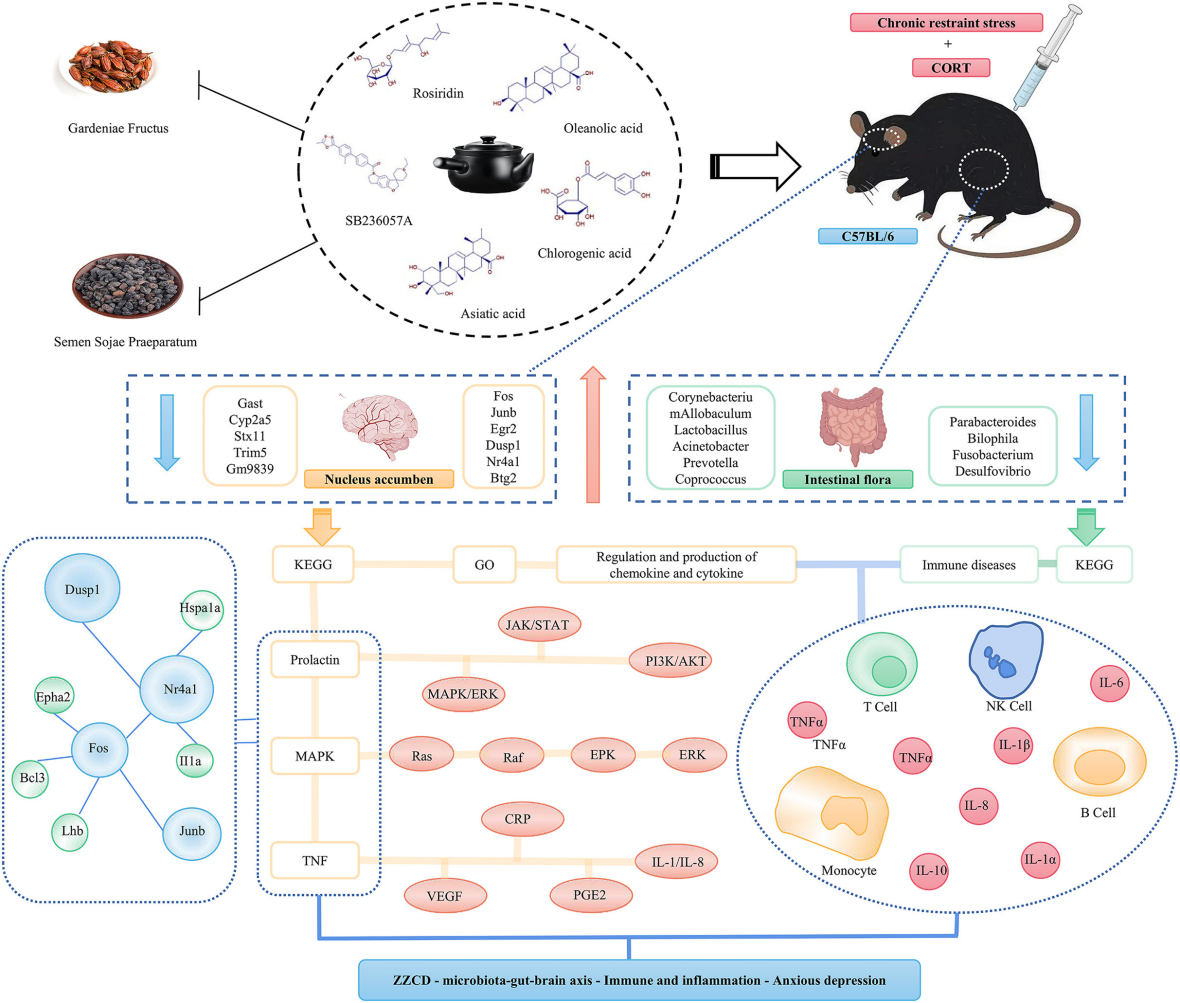
ZZCD ameliorates microbiota–gut–brain axis in mice with anxiety depression. ZZCD exerts effect on anxiety depression by regulating the intestinal flora, participating in the process of neuroactive ligand/receptor interaction, regulating the secretion of prolactin and estrogen, and interfering with MAPK and TNF signaling pathways to reduce inflammation levels.

In this study, the original dose and origin of Chinese medicinals in the standard decoction of ZZCD were determined. Subsequently, the chemical ingredients of ZZCD standard decoction and its effects on behavior as well as on the levels of inflammatory factors, neurotransmitters and HPA axis hormones in peripheral blood, intestinal flora and NAc of anxious depression mice were investigated. ZZCD exerts pleiotropic antidepressant effects by regulating HPA axis and gut microbiota, participating in the process of neuroactive ligand/receptor interaction, regulating the secretion of prolactin and estrogen, and interfering with MAPK and TNF signaling pathways to reduce inflammation levels (Figure 10). Therefore, it is possible that ZZCD standard decoction could be further developed to be a novel antidepressant with strong effect and high safety.

## AUTHOR CONTRIBUTIONS

K. M., and X. B. S. conceived, designed, and coordinated the study. Q. C. M., F. T. and G. Y. W. performed the data analysis. X. H. T., X. Y. X., J. P. and D. J. G. performed the experiments. K. M. and Z. Z. wrote and revised the manuscript. All the authors reviewed, edited, and approved the final manuscript.

## FUNDING INFORMATION

This study was granted by National Nature Science Foundation of China (81903948 and 82204795), Shandong Provincial Natural Science Foundation (ZR2023QH052). Furthermore, it was also supported by special fund for high‐level talent cultivation project of traditional Chinese medicine in Shandong Province (2023–143), Shandong Province Universities' Development Plan for Youth Innovation Teams (2019–9‐202), Shanghai Science and Technology Development Foundation (2023–761027) and Shandong Students' Platform for Innovation and Entrepreneurship Training Program (202210441006 and 2023014).

## CONFLICT OF INTEREST STATEMENT

The authors declare that the research was conducted in the absence of any commercial or financial relationships that could be construed as a potential conflict of interest.

## Supporting information


Data S1.

## Data Availability

The raw data supporting the conclusions of this manuscript will be made available by the authors, without undue reservation, to any qualified researcher. The datasets presented in this study can be found in online database. The mass spectrometry proteomics data have been deposited to the ProteomeXchange Consortium (http://proteomecentral.proteomexchange.org) with the dataset identifier PXD040673. The database name and accession number are: NCBI Sequence Read Archive (SRA) and accession number PRJNA938739, respectively.
