# Supplementary material for: Zhi Zi Chi decoction (Gardeniae fructus and semen Sojae Praeparatum) attenuates anxious depression via modulating microbiota–gut–brain axis in corticosterone combined with chronic restraint stress‐induced mice
Source: CNS Neurosci Ther. 2023 Oct 31;30(4):e14519. doi: 10.1111/cns.14519 (PMC11017446; doi:10.1111/cns.14519)
Supplement: Supplementary file 1 — Data S1. [file CNS-30-e14519-s001.docx]

Supplementary Material

Zhi Zi Chi Decoction (Gardeniae Fructus and Semen Sojae Praeparatum) attenuates anxious depression via modulating microbiota-gut-brain axis in *corticosterone combined with chronic restraint stress*-induced mice

Xuanhe Tian ^1^ ^#^, Guangyan Wang ^2#^, Fei Teng ^1 #^, Xiaoyan Xue ^1^, Jin Pan ^1^, Qiancheng Mao ^1^, Dongjing Guo ^1^, Xiaobin Song ^1,3 *^ and Ke Ma ^3 *^

*** Correspondence:**

Dr. Ke Ma, make19880710@163.com；Dr. Xiaobin Song, xiaobin_song.com@163.com.


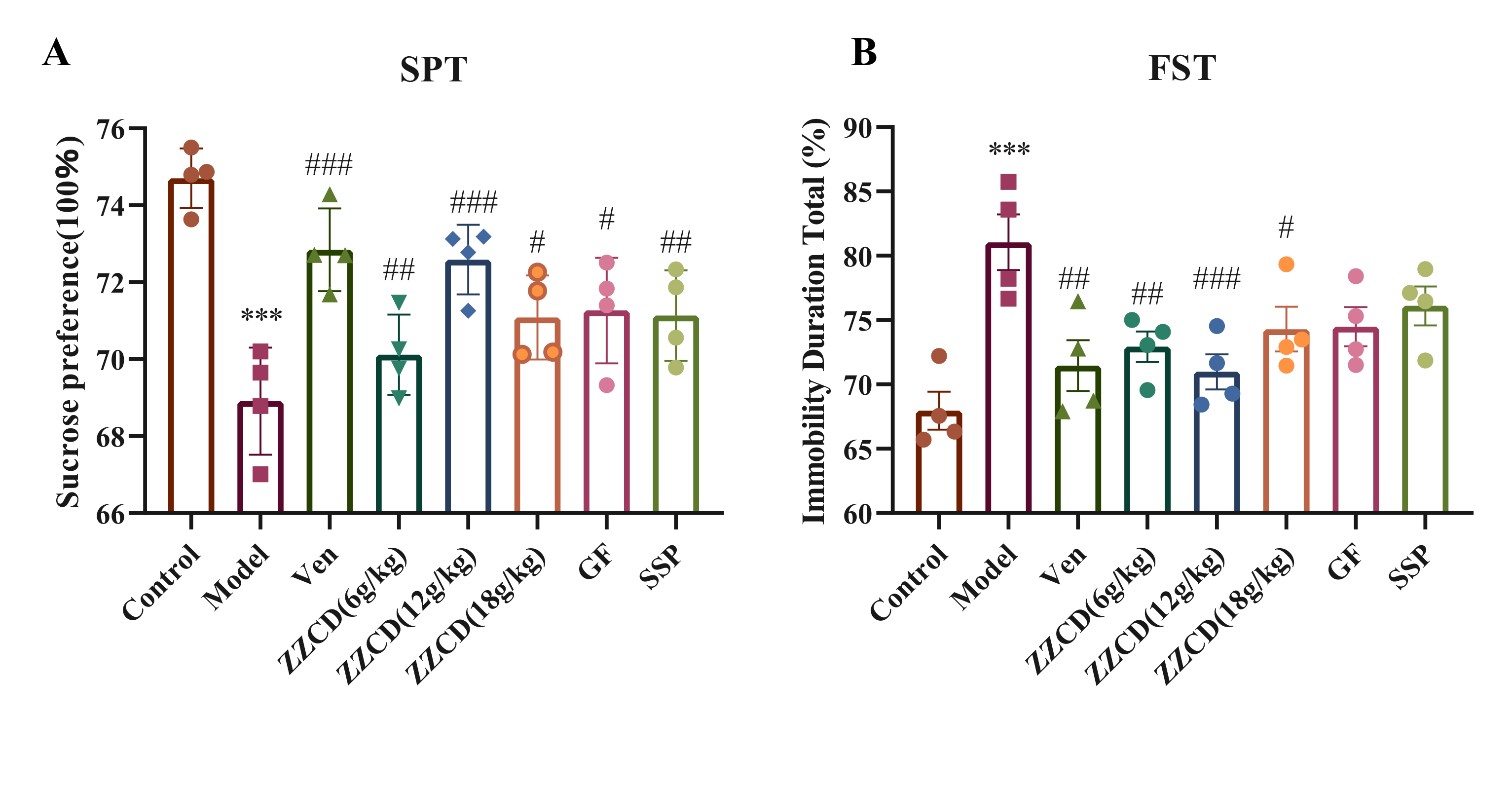


**Supplementary Figure 1. Comparison of different concentrations of ZZCD, SSP decoction and GF decoction on anxious depression.**


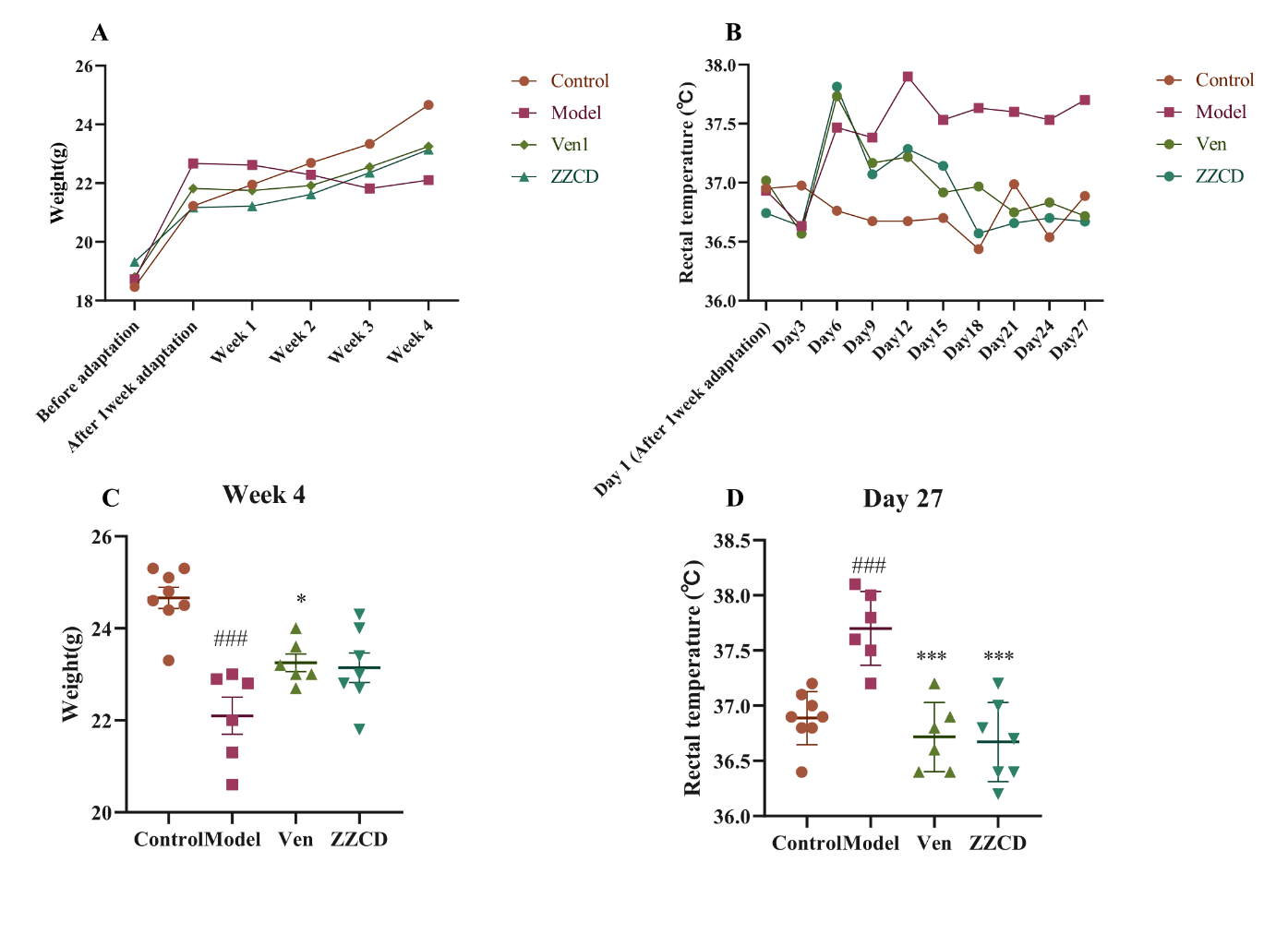


**Supplementary Figure 2. Body weights and rectal temperature of mice**


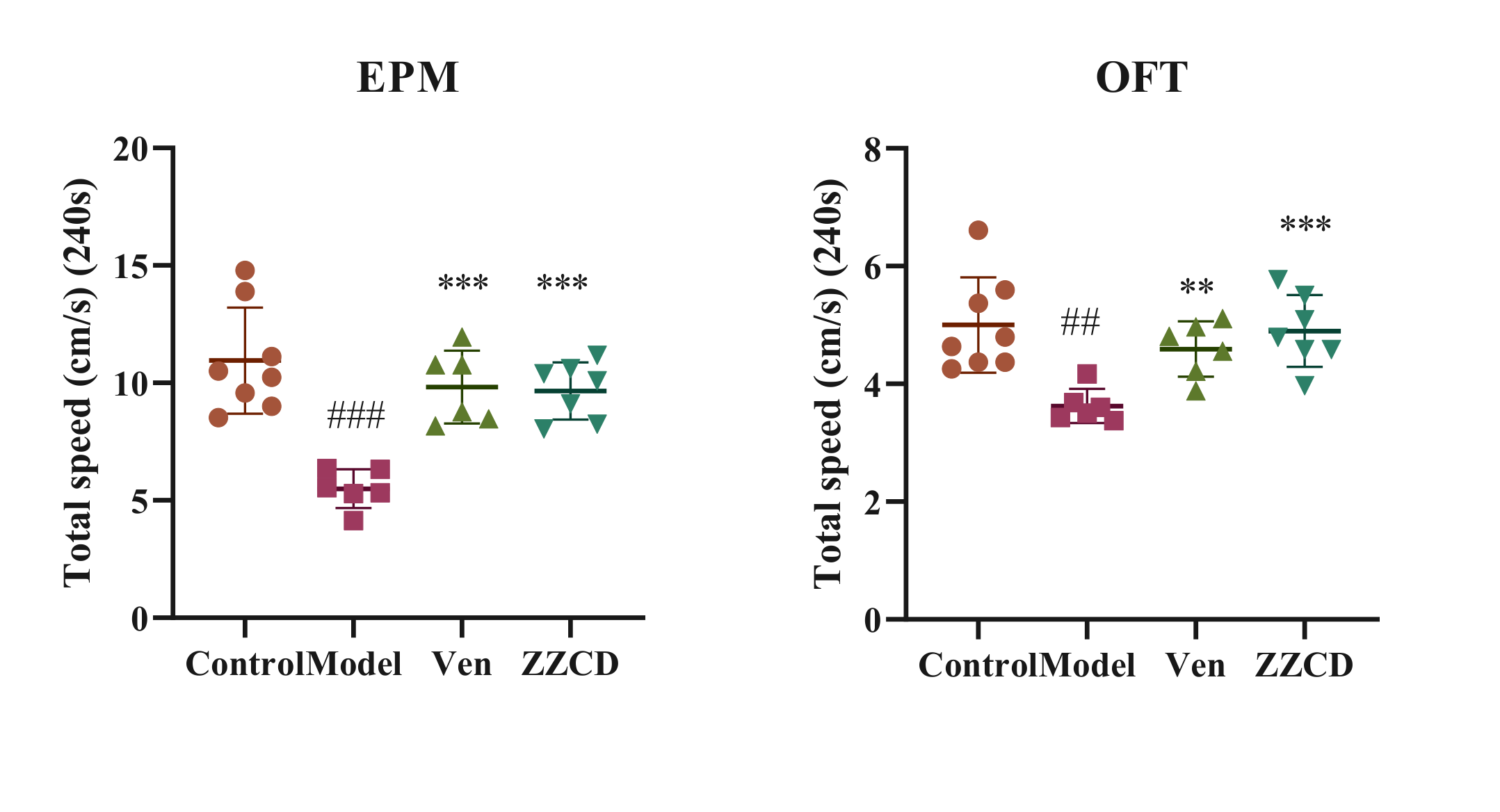


**Supplementary Figure 3. Total speed in EPM and OFT.**

**
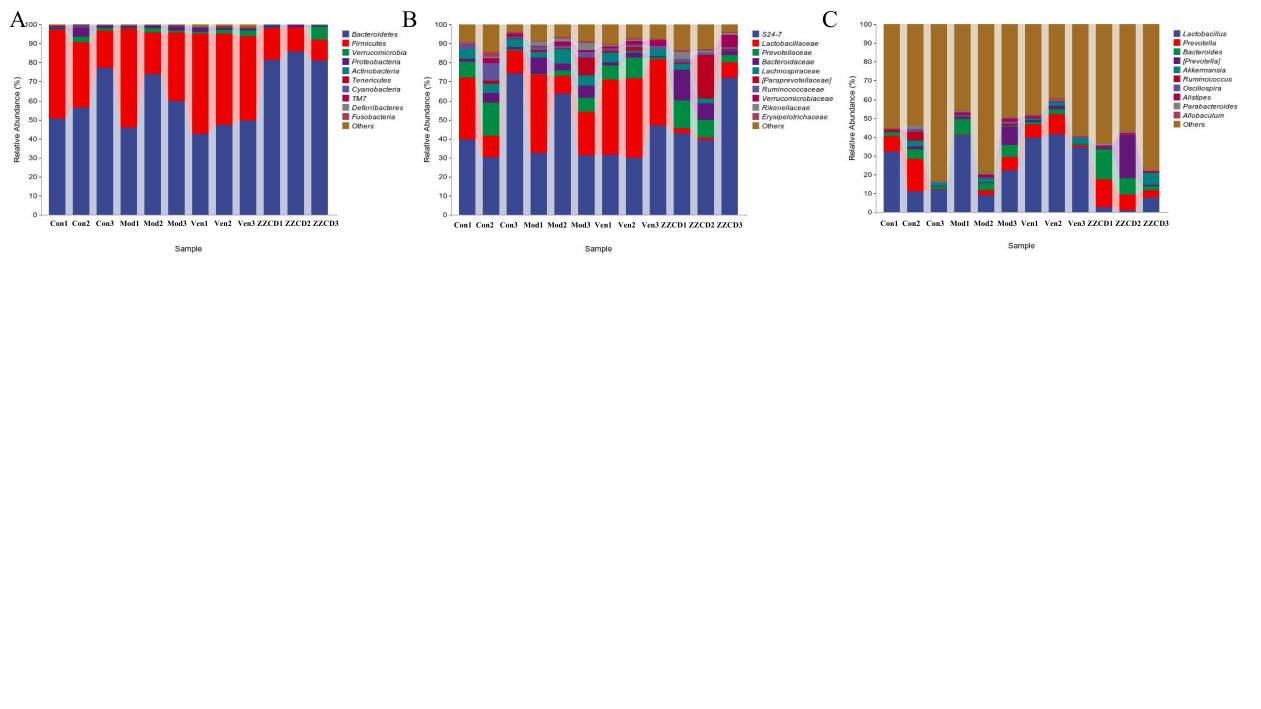
**

**Supplementary Figure 4.** **Bar graphs of relative abundance of species at (A) phylum, (B) family, and (C) genus levels in samples from the blank Control, Model, Ven, and ZZCD.**

**
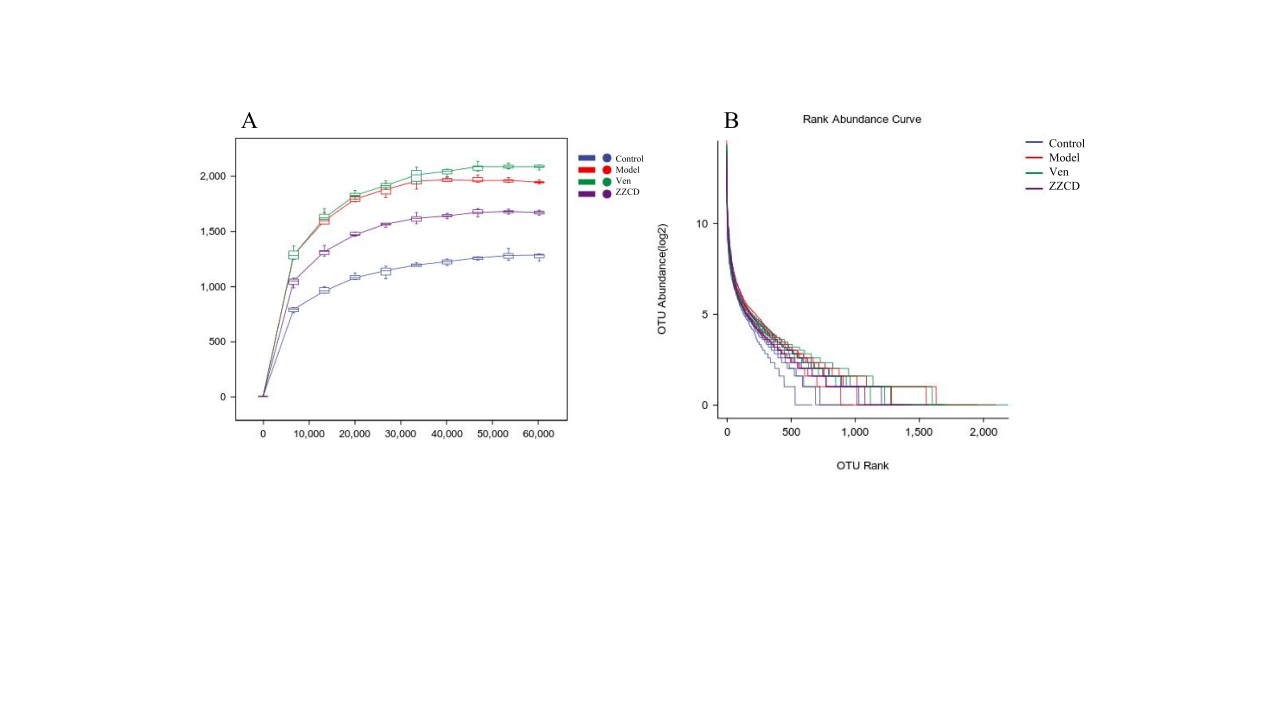
**

**Supplementary Figure 5.** **Sample rarefaction curve (A) and rank abundance curve (B) of Control, Model, Ven, and ZZCD.**

**
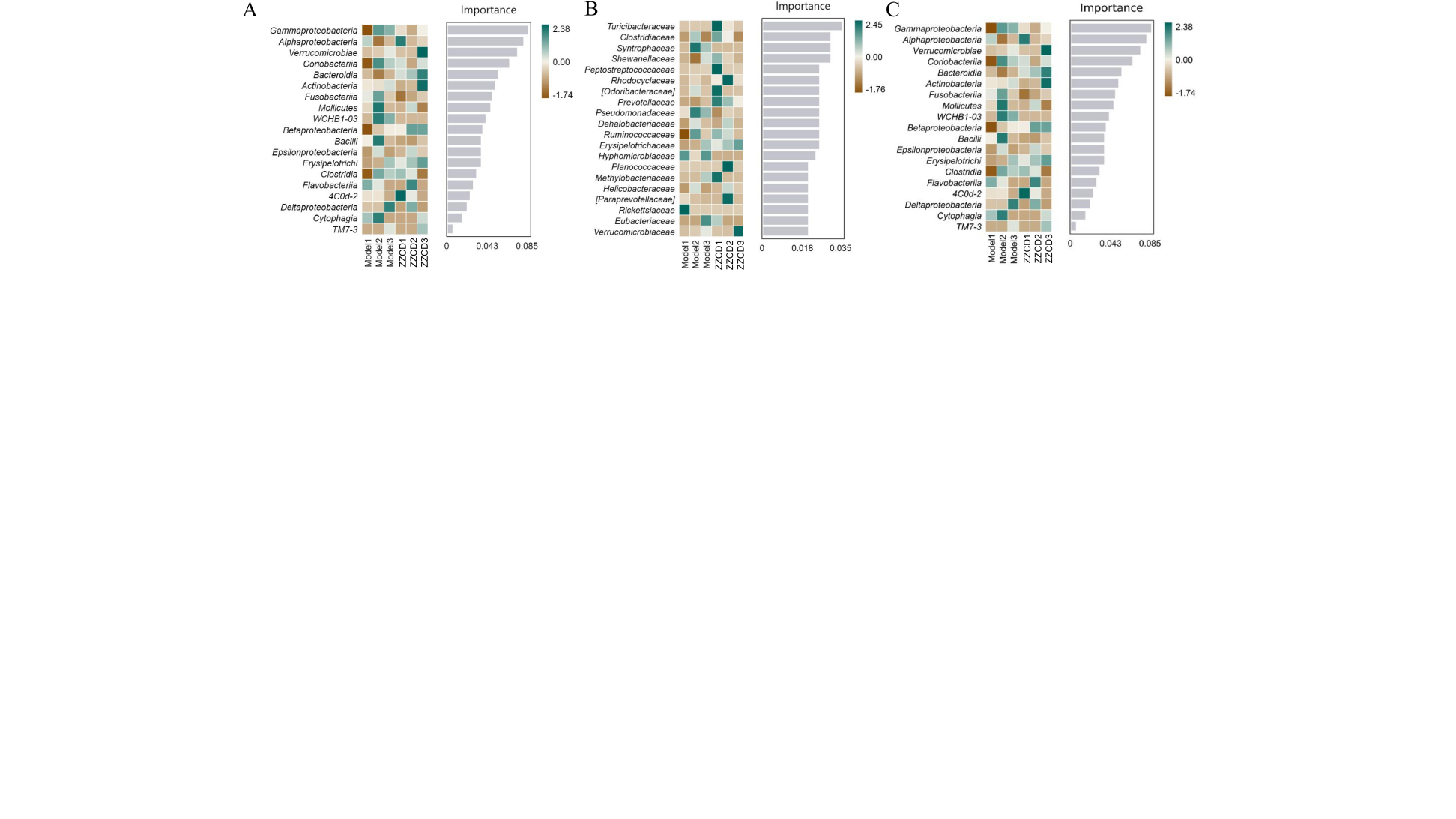
**

**Supplementary Figure 6. Random forest analysis of the flora at the phylum (A), family (B) and genus (C) levels in Model and ZZCD.**


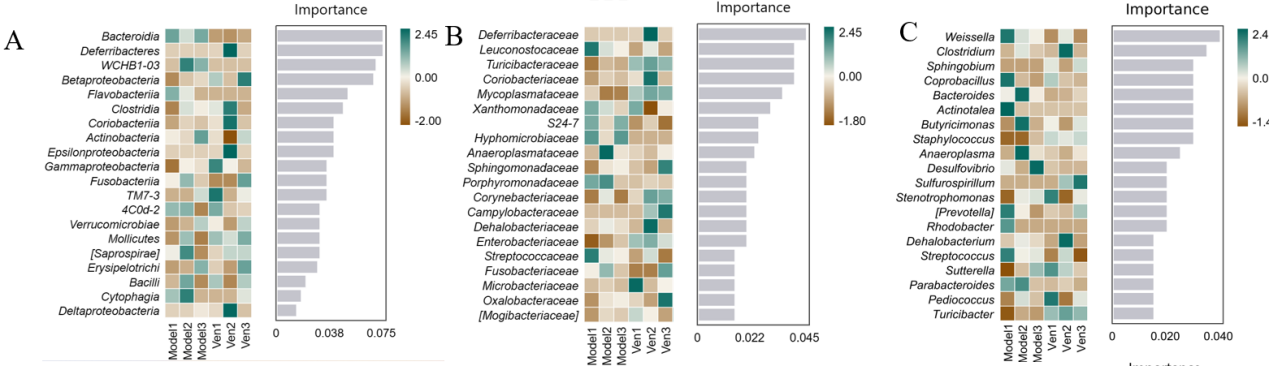


**Supplementary Figure 7.**  **Random forest analysis of the flora at the phylum (A), family (B) and genus (C) levels in the Model and Ven.**

**
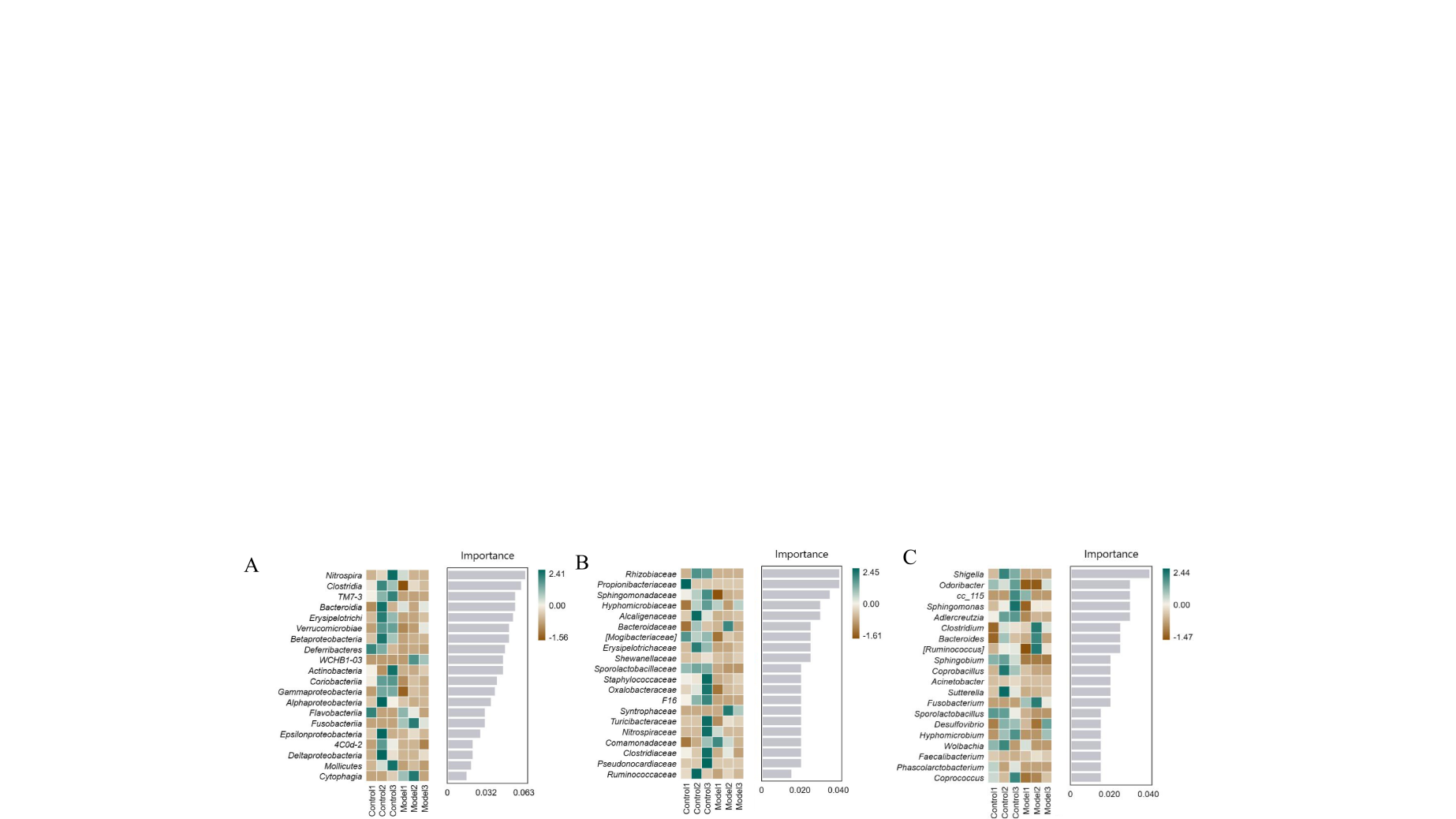
**

**Supplementary Figure 8.**  **Random forest analysis of the flora at the phylum (A), family (B) and genus (C) levels in the Control and Model.**

**
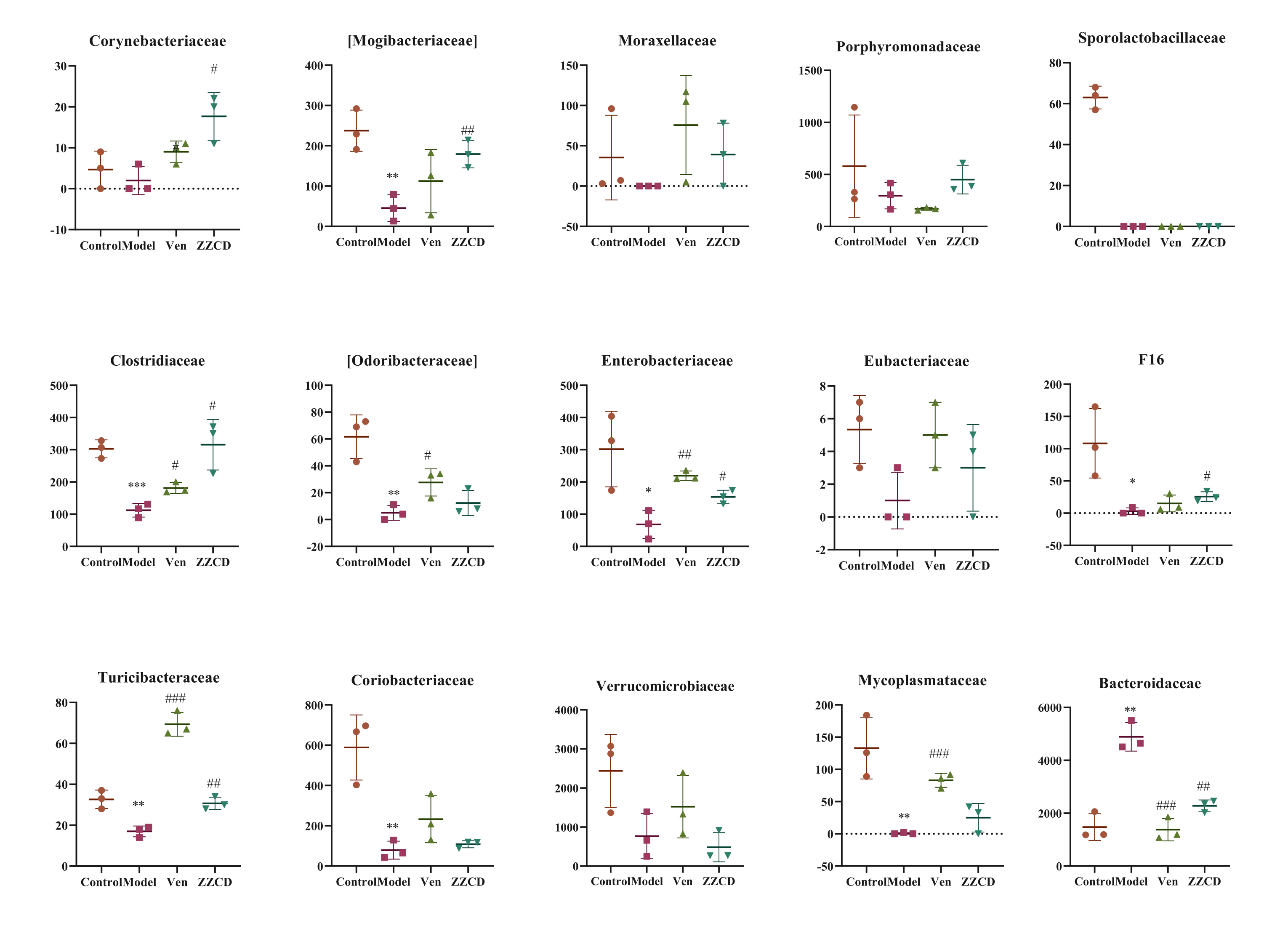
**

**Supplementary Figure 9. Histogram of the differences in the abundance of bacterial populations at the family level.**Data are expressed as mean ± SEM (n=3-4 per group), ^*^*P*<0.05, ^**^*P*<0.01, and ^***^*P*<0.001 *vs* the Control group; ^#^*P*<0.05, ^##^*P*<0.01, and ^###^*P*<0.001 *vs* the Model group. Model: CORT combined with chronic restraint stress+ Saline, ZZCD: CORT combined with chronic restraint stress+ZZCD treatment.

**Table S1.** **Gene primer sequence information.**

| **Gene** | **primer sequence** |
| --- | --- |
| Dusp1 | 5′- GCGCTCCACTCAAGTCTTCT-3′  5′-AGAGGGGTACTACAGGAGCT-3′ |
| Nr4a1 | 5′-CTGCCTTCCTGGAACTCTTCA-3′  5′-CGGGTTTAGATCGGTATGCC-3′ |
| β-actin | 5′- GCTTCTTTGCAGCTCCTTCGT-3′  5′- ATATCGTCATCCATGGCGAAC-3′ |

**Table S2. Characterization of the chemical constituents in GF standard decoction by LC-MS.**

| Number | Name | Model | RT [min] | Formula | m/z |
| --- | --- | --- | --- | --- | --- |
| 1 | L-Glutamic acid | - | 1.305 | C5 H9 N O4 | 268.10388 |
| 2 | D-Glucose 6-phosphate | - | 1.84 | C6 H13 O9 P | 259.02231 |
| 3 | Uridine 5'-diphosphogalactose | - | 4.209 | C15 H24 N2 O17 P2 | 565.0481 |
| 4 | trans-3-Indoleacrylic acid | - | 7.329 | C11 H9 N O2 | 188.0705 |
| 5 | D-(+)-Tryptophan | - | 7.331 | C11 H12 N2 O2 | 203.08189 |
| 6 | Gentisic acid | - | 7.386 | C7 H6 O4 | 153.01816 |
| 7 | 2-[3-(tert-butyl)-1-(4-fluorobenzyl)-1H-pyrazol-5-yl]-5-(2-thienyl)-1,3,4-oxadiazole | - | 7.62 | C20 H19 F N4 O S | 383.13068 |
| 8 | Neochlorogenic acid | - | 8.309 | C16 H18 O9 | 353.08768 |
| 9 | Perillic acid | - | 8.58 | C10 H14 O2 | 165.09093 |
| 10 | N4-[2-(2-pyridyl)ethyl]-1-(4-chlorophenyl)-5-propyl-1H-pyrazole-4-carboxamide | - | 9.695 | C20 H21 Cl N4 O | 369.15155 |
| 11 | 7-hydroxy-6-methoxy-2H-chromen-2-one | - | 9.724 | C10 H8 O4 | 193.04956 |
| 12 | N-[(1R,9S)-6-Oxo-11-(2-pyrimidinyl)-7,11-diazatricyclo[7.3.1.02,7]trideca-2,4-dien-5-yl]isonicotinamide | - | 9.729 | C21 H20 N6 O2 | 371.16718 |
| 13 | (3R,4R)-4-[(3,4-dimethoxyphenyl)methyl]-3-[(4-hydroxy-3-methoxyphenyl)methyl]oxolan-2-one | - | 10.447 | C21 H24 O6 | 411.12534 |
| 14 | Geniposide | - | 10.496 | C17 H24 O10 | 433.13428 |
| 15 | 4-Hydroxybenzylalcohol | - | 10.499 | C7 H8 O2 | 123.04372 |
| 16 | methyl (1S,4aS,7aS)-7-(hydroxymethyl)-1-{[(2S,3R,4S,5S,6R)-3,4,5-trihydroxy-6-({[(2R,3R,4S,5S,6R)-3,4,5-trihydroxy-6-({[(2E)-3-(4-hydroxyphenyl)prop-2-enoyl]oxy}methyl)oxan-2-yl]oxy}methyl)oxan-2-yl]oxy}-1H,4aH,5H,7aH-cyclopenta[c]pyran-4-carboxylate | - | 13.116 | C32 H40 O17 | 714.25873 |
| 17 | Estrone | - | 14.02 | C18 H22 O2 | 249.06677 |
| 18 | 3,5-di-tert-Butyl-4-hydroxybenzaldehyde | - | 17.996 | C15 H22 O2 | 235.16876 |
| 19 | Palmitoleic acid | - | 21.967 | C16 H30 O2 | 253.21724 |
| 20 | Stearic acid | - | 24.047 | C18 H36 O2 | 283.26401 |
| 21 | Betaine | + | 1.393 | C5 H11 N O2 | 118.08645 |
| 22 | 1-Aminocyclohexanecarboxylic acid | + | 1.648 | C7 H13 N O2 | 144.1019 |
| 23 | Adenosine | + | 4.719 | C10 H13 N5 O4 | 295.11316 |
| 24 | Geniposidic acid | + | 7.881 | C16 H22 O10 | 373.11374 |
| 25 | Methyl 1-(hexopyranosyloxy)-5-hydroxy-7-(hydroxymethyl)-1,4a,5,7a-tetrahydrocyclopenta[c]pyran-4-carboxylate | + | 7.887 | C17 H24 O11 | 449.12991 |
| 26 | Caprolactam | + | 7.96 | C6 H11 N O | 114.09164 |
| 27 | 7,8-Dihydroxy-4-methylcoumarin | + | 8.286 | C23 H23 Cl N2 O2 S | 427.12045 |
| 28 | (2-Chlorophenyl){2-[(2-methoxyphenyl)imino]-4-methylidene-3-thia-1-azaspiro[4.5]dec-1-yl}methanone | + | 8.286 | C10 H8 O4 | 175.03883 |
| 29 | Methyl 1-(hexopyranosyloxy)-7-hydroxy-7-(hydroxymethyl)-1,4a,7,7a-tetrahydrocyclopenta[c]pyran-4-carboxylate | + | 8.288 | C17 H24 O11 | 449.12946 |
| 30 | 6,7-Dihydroxy-4-methylcoumarin | + | 8.67 | C10 H8 O4 | 225.07547 |
| 31 | Mussaenosidic acid | + | 8.78 | C16 H24 O10 | 375.12955 |
| 32 | Shanzhiside methyl ester | + | 9.805 | C17 H26 O11 | 389.14337 |
| 33 | 1,2,3,4-Tetramethyl-1,3-cyclopentadiene | + | 11.058 | C9 H14 | 123.11695 |
| 34 | Sinapinic acid | + | 11.985 | C11 H12 O5 | 207.06506 |
| 35 | 4,5-Dicaffeoylquinic acid | + | 13.441 | C25 H24 O12 | 515.11938 |
| 36 | 3,4,5-trihydroxy-6-({[3,4,5-trihydroxy-6-(hydroxymethyl)oxan-2-yl]oxy}methyl)oxan-2-yl 2,6,6-trimethylcyclohex-1-ene-1-carboxylate | + | 13.482 | C22 H36 O12 | 510.254 |
| 37 | Azelaic acid | + | 13.495 | C9 H16 O4 | 187.09669 |
| 38 | Isophorone | + | 13.883 | C9 H14 O | 139.11162 |
| 39 | 2-[(2S,3R,4S,5R)-3,4-Dihydroxy-5-{[(isopropylcarbamoyl)amino]methyl}tetrahydro-2-furanyl]-N-[2-(dimethylamino)ethyl]acetamide | + | 14.714 | C15 H30 N4 O5 | 381.19193 |
| 40 | 3-tert-Butyladipic acid | + | 14.727 | C10 H18 O4 | 201.11258 |
| 41 | N-Butylbenzenesulfonamide | + | 14.755 | C10 H15 N O2 S | 214.08929 |
| 42 | 4,4-Diphenylmethane diisocyanate | + | 15.918 | C15 H10 N2 O2 | 293.15302 |
| 43 | Cuminaldehyde | + | 17.223 | C10 H12 O | 149.09581 |
| 44 | (±)9-HpODE | + | 18.271 | C18 H32 O4 | 311.22302 |
| 45 | Dipropyleneglycol dibenzoate | + | 18.276 | C20 H22 O5 | 365.13516 |
| 46 | Diisobutylphthalate | + | 18.626 | C16 H22 O4 | 301.14044 |
| 47 | Sphingosine (d18:1) | + | 19.763 | C18 H37 N O2 | 322.27121 |
| 48 | 18-β-Glycyrrhetinic acid | + | 20.735 | C17 H34 O4 | 325.23422 |
| 49 | 2,3-dihydroxypropyl 12-methyltridecanoate | + | 20.735 | C30 H46 O4 | 471.34628 |
| 50 | 1-Stearoylglycerol | + | 23.125 | C21 H42 O4 | 381.29633 |
| 51 | Diisooctyl phthalate | + | 23.137 | C24 H38 O4 | 413.26529 |
| 52 | Dodecamethylcyclohexasiloxane | + | 24.528 | C12 H36 O6 Si6 | 445.11914 |

**Table S3. Characterization of the chemical constituents in SSP standard decoction by LC-MS.**

| Number | Name | Modle | RT [min] | Formula | m/z |
| --- | --- | --- | --- | --- | --- |
| 1 | L-Histidine | - | 1.044 | C6 H9 N3 O2 | 154.0611 |
| 2 | Betaine | - | 1.378 | C5 H11 N O2 | 118.08643 |
| 3 | Gluconic acid | - | 1.4 | C6 H12 O7 | 195.05023 |
| 4 | L-Tyrosine | - | 2.925 | C9 H11 N O3 | 182.08131 |
| 5 | Xanthine | - | 3.694 | C5 H4 N4 O2 | 151.02509 |
| 6 | Glycyl-L-leucine | - | 4.894 | C8 H16 N2 O3 | 189.12344 |
| 7 | Mesaconic acid | - | 4.963 | C5 H6 O4 | 129.01816 |
| 8 | Xanthosine | - | 6.363 | C10 H12 N4 O6 | 283.06873 |
| 9 | Oregonin | - | 6.51 | C24 H30 O10 | 477.17282 |
| 10 | Caprolactam | - | 7.983 | C6 H11 N O | 114.09176 |
| 11 | 6-Methoxyquinoline | - | 9.451 | C10 H9 N O | 160.07567 |
| 12 | Isophthalic acid | - | 10.134 | C8 H6 O4 | 165.01849 |
| 13 | 4-(hydroxymethyl)benzoic acid | - | 10.226 | C8 H8 O3 | 151.03894 |
| 14 | 4-Methoxycinnamic acid | - | 10.266 | C10 H10 O3 | 161.05962 |
| 15 | 5,7-Dihydroxy-2-(4-hydroxyphenyl)-6,8-bis[3,4,5-trihydroxy-6-(hydroxymethyl)tetrahydro-2H-pyran-2-yl]-4H-chromen-4-one | - | 10.958 | C27 H30 O15 | 593.15143 |
| 16 | Apigetrin | - | 12.248 | C21 H20 O10 | 477.10376 |
| 17 | Ethylphthalyl ethylglycolate | - | 13.059 | C14 H16 O6 | 281.10178 |
| 18 | Acridine | - | 13.114 | C13 H9 N | 180.08066 |
| 19 | Daidzein | - | 14.17 | C15 H10 O4 | 253.05028 |
| 20 | 1-(3-methoxy-4-{[(2S,3R,4S,5S,6R)-3,4,5-trihydroxy-6-({[(2R,3R,4R,5R,6S)-3,4,5-trihydroxy-6-methyloxan-2-yl]oxy}methyl)oxan-2-yl]oxy}phenyl)ethan-1-one | - | 15.035 | C21 H30 O12 | 513.13629 |
| 21 | 2,4-Dimethylbenzaldehyde | - | 15.064 | C9 H10 O | 135.0804 |
| 22 | 2,2,6,6-Tetramethyl-1-piperidinol (TEMPO) | - | 15.725 | C9 H19 N O | 158.15387 |
| 23 | 4,4-Diphenylmethane diisocyanate | - | 15.923 | C15 H10 N2 O2 | 249.0667 |
| 24 | Arjungenin | - | 16.566 | C30 H48 O6 | 503.33844 |
| 25 | Chrysin | - | 16.591 | C15 H10 O4 | 255.06483 |
| 26 | Glycitein | - | 16.943 | C16 H12 O5 | 285.07544 |
| 27 | Apigenin | - | 19.056 | C15 H10 O5 | 269.0455 |
| 28 | 18-β-Glycyrrhetinic acid | - | 20.401 | C30 H46 O4 | 469.33252 |
| 29 | 2-(14,15-Epoxyeicosatrienoyl) glycerol | - | 21.521 | C23 H38 O5 | 377.26556 |
| 30 | Palmitoyl ethanolamide | - | 21.527 | C18 H37 N O2 | 300.2894 |
| 31 | Oleic acid alkyne | - | 21.566 | C18 H30 O2 | 277.21741 |
| 32 | Palmitic acid | - | 22.725 | C16 H32 O2 | 255.23274 |
| 33 | Stearic acid | - | 24.068 | C18 H36 O2 | 283.26428 |
| 34 | SB236057A | - | 25.097 | C33 H34 N4 O3 | 535.2699 |
| 35 | DL-Homoserine | + | 1.264 | C4 H9 N O3 | 120.06574 |
| 36 | L-Glutamic acid | + | 1.272 | C5 H9 N O4 | 148.06027 |
| 37 | L-Aspartic acid | + | 1.274 | C4 H7 N O4 | 134.04482 |
| 38 | D-(+)-Proline | + | 1.416 | C5 H9 N O2 | 116.07086 |
| 39 | Trigonelline | + | 1.425 | C7 H7 N O2 | 138.05495 |
| 40 | Adenine | + | 1.468 | C5 H5 N5 | 136.06197 |
| 41 | L-Isoleucine | + | 2.581 | C6 H13 N O2 | 132.10191 |
| 42 | 4-Oxoproline | + | 2.646 | C5 H7 N O3 | 128.03413 |
| 43 | UDP-N-acetylglucosamine | + | 4.652 | C17 H27 N3 O17 P2 | 606.07458 |
| 44 | Adenosine | + | 4.757 | C10 H13 N5 O4 | 268.10388 |
| 45 | Pseudoephedrine | + | 5.17 | C10 H15 N O | 166.12265 |
| 46 | N-Methyl-2-AI | + | 5.171 | C10 H13 N | 148.11203 |
| 47 | Leucylproline | + | 5.592 | C11 H20 N2 O3 | 229.15469 |
| 48 | 3'-Adenosine monophosphate (3'-AMP) | + | 5.915 | C10 H14 N5 O7 P | 348.06995 |
| 49 | Guanosine monophosphate (GMP) | + | 5.932 | C10 H14 N5 O8 P | 362.05087 |
| 50 | Pseudoephedrine | + | 5.967 | C10 H15 N O | 148.11197 |
| 51 | 2-(2-amino-3-methylbutanamido)-3-phenylpropanoic acid | + | 8.023 | C14 H20 N2 O3 | 265.15463 |
| 52 | N-Acetyl-DL-norvaline | + | 8.389 | C7 H13 N O3 | 158.08128 |
| 53 | 4-hydroxy-5,8-dimethylquinoline-3-carboxylic acid | + | 9.892 | C12 H11 N O3 | 218.08109 |
| 54 | 2-AI | + | 10.897 | C9 H11 N | 134.09637 |
| 55 | Daidzin | + | 11.309 | C21 H20 O9 | 417.1174 |
| 56 | D(+)-Phenyllactic acid | + | 11.396 | C9 H10 O3 | 165.05479 |
| 57 | Propranolol | + | 13.802 | C16 H21 N O2 | 260.16425 |
| 58 | (±)-Abscisic acid | + | 13.821 | C15 H20 O4 | 263.12915 |
| 59 | 4-(tert-butyl)phenyl 3,5-dimethylisoxazole-4-carboxylate | + | 14.05 | C16 H19 N O3 | 274.14349 |
| 60 | Genistein | + | 15.026 | C15 H10 O5 | 269.04538 |
| 61 | N,N'-Dicyclohexylurea | + | 16.329 | C13 H24 N2 O | 225.19571 |
| 62 | β-Asarone | + | 16.337 | C12 H16 O3 | 209.11696 |
| 63 | BB-22 3-Carboxyindole metabolite | + | 17.204 | C16 H19 N O2 | 258.14841 |
| 64 | Cuminaldehyde | + | 17.246 | C10 H12 O | 149.09586 |
| 65 | Bis(4-ethylbenzylidene)sorbitol | + | 17.248 | C24 H30 O6 | 415.21045 |
| 66 | 2,4-Dimethylbenzaldehyde | + | 17.248 | C9 H10 O | 135.08023 |
| 67 | (3β,5ξ,9ξ)-3,23-Dihydroxy-1-oxoolean-12-en-28-oic acid | + | 17.655 | C30 H46 O5 | 485.32742 |
| 68 | Propranolol | + | 17.67 | C16 H21 N O2 | 260.16388 |
| 69 | Diisobutylphthalate | + | 18.641 | C16 H22 O4 | 301.14038 |
| 70 | 4-[3-(benzylamino)butyl]-2-methoxyphenol | + | 18.685 | C18 H23 N O2 | 286.17972 |
| 71 | 4-[5-(5-anilino-1,2,4-thiadiazol-3-yl)-4-methylpyrimidin-2-yl]benzamide | + | 19.235 | C20 H16 N6 O S | 411.09296 |
| 72 | Soyasaponin I | + | 19.242 | C48 H78 O18 | 941.51105 |
| 73 | 2-(14,15-Epoxyeicosatrienoyl) glycerol | + | 19.289 | C23 H38 O5 | 395.27612 |
| 74 | 2,3-dihydroxypropyl 12-methyltridecanoate | + | 20.751 | C17 H34 O4 | 325.2345 |
| 75 | Octadecyl 3-(3,5-di-tert-butyl-4-hydroxyphenyl)propionate | + | 21.817 | C35 H62 O3 | 548.50299 |
| 76 | ?-Linolenic Acid | + | 21.966 | C18 H30 O2 | 277.21744 |
| 77 | Palmitoleic acid | + | 21.972 | C16 H30 O2 | 253.21729 |
| 78 | Linoleic acid | + | 22.216 | C18 H32 O2 | 279.233 |
| 79 | Oleanolic acid | + | 22.242 | C30 H48 O3 | 439.35645 |
| 80 | 1-Stearoylglycerol | + | 23.128 | C21 H42 O4 | 381.29636 |
| 81 | Diisooctyl phthalate | + | 23.137 | C24 H38 O4 | 391.28333 |
| 82 | Dodecamethylcyclohexasiloxane | + | 24.54 | C12 H36 O6 Si6 | 445.11911 |

**Table S4. The body weight and rectal temperature of the mice**

| number | Weight (g) | | | | | | Rectal temperature (℃) | | | | | | | | | |
| --- | --- | --- | --- | --- | --- | --- | --- | --- | --- | --- | --- | --- | --- | --- | --- | --- |
|  | Before adaptation | After 1week adaptation | Week 1 | Week 2 | Week 3 | Week 4 | Day 1 (After 1week adaptation) | Day 3 | Day 6 | Day 9 | Day 12 | Day 15 | Day 18 | Day 21 | Day 24 | Day 27 |
| Control1 | 19.2 | 20.7 | 21.1 | 22.9 | 23.9 | 25.3 | 37 | 36.9 | 36.5 | 36.6 | 37.1 | 36.7 | 36.7 | 37 | 36.8 | 37.1 |
| Control2 | 18.1 | 22 | 22.3 | 23.1 | 23.5 | 24.8 | 37.1 | 37.3 | 37 | 36.8 | 36.6 | 37.1 | 36.1 | 36.4 | 36.5 | 36.8 |
| Control3 | 17.7 | 20.9 | 22 | 22.3 | 22.8 | 24.6 | 37.1 | 36.4 | 36.6 | 36.8 | 37.1 | 36.8 | 36.1 | 37.2 | 36.5 | 36.9 |
| Control4 | 18.1 | 19.2 | 20 | 21.4 | 22.5 | 25.1 | 37.1 | 36.7 | 36.9 | 36 | 36.6 | 36.1 | 37 | 37.3 | 37.2 | 37 |
| Control5 | 20.1 | 21 | 22.3 | 23 | 24.3 | 25.3 | 36.8 | 37.5 | 37.1 | 36.4 | 36.2 | 37.2 | 36.1 | 37 | 36.9 | 37.2 |
| Control6 | 17.9 | 20.5 | 21.5 | 21.7 | 22 | 23.3 | 36.9 | 36.5 | 36.6 | 36.9 | 37.1 | 36.3 | 36.4 | 37.1 | 36.1 | 36.8 |
| Control7 | 18.8 | 22.5 | 23 | 23.3 | 23.7 | 24.4 | 36.5 | 37.3 | 36.6 | 37 | 36.2 | 36.8 | 36 | 37.2 | 36 | 36.4 |
| Control8 | 17.8 | 23 | 23.4 | 23.8 | 24 | 24.5 | 37.1 | 37.2 | 36.8 | 36.9 | 36.5 | 36.6 | 37.1 | 36.7 | 36.3 | 36.9 |
| Model1 | 17.9 | 21.3 | 21.5 | 20.8 | 20.6 | 20.6 | 37 | 36.5 | 37.8 | 37.1 | 38 | 37.9 | 38.1 | 37.3 | 37.2 | 38.1 |
| Model2 | 19.4 | 22.8 | 23 | 23.1 | 22.8 | 23 | 37 | 36.4 | 37.1 | 37.2 | 38.2 | 37.6 | 37.9 | 38.2 | 37.2 | 37.5 |
| Model3 | 17.9 | 23.1 | 23 | 23.2 | 22.8 | 22.9 | 36.9 | 36.6 | 38 | 36.9 | 37.3 | 37.2 | 37.5 | 37.9 | 38.3 | 37.8 |
| Model4 | 19.5 | 23.7 | 24.1 | 23.5 | 22 | 21.3 | 36.8 | 36.9 | 37 | 38.3 | 37.5 | 37.4 | 36.9 | 36.9 | 37.5 | 37.2 |
| Model5 | 18.7 | 23 | 23.5 | 23.1 | 23 | 22.8 | 37.2 | 37 | 38 | 36.9 | 38.3 | 36.9 | 37.1 | 37.4 | 37.9 | 38 |
| Model6 | 19 | 22.1 | 20.6 | 20 | 19.7 | 22 | 36.7 | 36.4 | 36.9 | 37.9 | 38.1 | 38.2 | 38.3 | 37.9 | 37.1 | 37.6 |
| Model7 | 18.6 | 20 | 19.1 | 19 | Died on Day 15 | | 36 | 36.5 | 37.6 | 38.1 | 38.5 |  |  |  |  |  |
| Model8 | 18.1 | 21.4 | 18.6 | Died on Day 8 | | | 37.3 | 36.3 | 37.5 |  |  |  |  |  |  |  |
| Ven1 | 18.6 | 22.4 | 22.5 | 22.2 | 23 | 23.2 | 37.3 | 36.7 | 38.2 | 37.4 | 37.4 | 36.4 | 36.4 | 36.9 | 37 | 36.4 |
| Ven2 | 18.9 | 21.8 | 22 | 22.3 | 22.8 | 23.6 | 37.4 | 36.6 | 36.9 | 37.2 | 37 | 36.9 | 37.3 | 36.3 | 37.3 | 37.2 |
| Ven3 | 18.6 | 20.1 | 20 | 21 | 22.1 | 23 | 37.3 | 36.3 | 38 | 37.1 | 36.6 | 37 | 36.6 | 36.8 | 36.7 | 36.6 |
| Ven4 | 19.3 | 23 | 23.3 | 23 | 22.2 | 22.7 | 36.6 | 37.1 | 38.2 | 36.9 | 37.6 | 37.2 | 37.2 | 36.7 | 36.4 | 36.9 |
| Ven5 | 19.4 | 21.6 | 21.1 | 22 | 23.4 | 24 | 36.8 | 36.4 | 37.7 | 37.3 | 37.3 | 37 | 37.3 | 37 | 37 | 36.8 |
| Ven6 | 18.6 | 23.1 | 23.2 | 22.3 | 21 | Died on Day 25 | 37.1 | 36.5 | 37.2 | 37.3 | 37.3 | 37.3 | 36.1 | 36.5 | 36.5 |  |
| Ven7 | 19.2 | 20.5 | 19.5 | 18.8 | Died on Day 16 | | 36.6 | 36.5 | 38 | 37.3 | 37.6 | 37.3 |  |  |  |  |
| Ven8 | 18 | 22 | 21.6 | 21 | 21.8 | 23 | 36.7 | 36.3 | 37.4 | 37.1 | 37.4 | 37 | 37 | 36.8 | 36.6 | 36.4 |
| ZZCD1 | 21.1 | 21.5 | 22 | 21.9 | 22.1 | 22.7 | 36.5 | 37 | 37.7 | 36.9 | 37.7 | 37 | 36.2 | 36.6 | 37.2 | 36.4 |
| ZZCD2 | 17.3 | 21 | 20.5 | 20 | 21 | 21.8 | 37.2 | 36.2 | 38 | 36.9 | 37.1 | 37.2 | 36.2 | 37.1 | 36.8 | 36.4 |
| ZZCD3 | 18.6 | 21.2 | 21 | 21.3 | 21.9 | 23 | 36.1 | 36.3 | 37.4 | 36.9 | 37 | 37.3 | 36.3 | 36.3 | 37.1 | 37.2 |
| ZZCD4 | 19.2 | 21.4 | 22 | 22.8 | 23.2 | Died on Day 24 | 36.5 | 36.5 | 37.7 | 37.4 | 36.6 | 37.1 | 36.8 | 36.3 |  |  |
| ZZCD5 | 19.6 | 22.1 | 21.9 | 22.5 | 23.1 | 24 | 36.6 | 36.3 | 38 | 36.9 | 37.1 | 37 | 37.3 | 36.2 | 36 | 37 |
| ZZCD6 | 19.1 | 20.9 | 21.1 | 22 | 22.9 | 23.4 | 36.7 | 36.5 | 38.3 | 37.3 | 37.1 | 37.2 | 37.3 | 36.1 | 36.8 | 36.8 |
| ZZCD7 | 19.5 | 20.4 | 20 | 21.2 | 22 | 22.8 | 37.2 | 37 | 37.1 | 37.5 | 37.4 | 37.2 | 36.3 | 37.3 | 36.9 | 36.2 |
| ZZCD8 | 20 | 21.1 | 22 | 22.4 | 23.5 | 24.3 | 36.9 | 37.1 | 38.2 | 37.1 | 37.6 | 37.1 | 36.4 | 37 | 36.1 | 36.7 |

**Table S5. The distance, time and speed of OFT and EPM.**

|  | OFT | | | | | | EPM | | | | | |
| --- | --- | --- | --- | --- | --- | --- | --- | --- | --- | --- | --- | --- |
| Number | Distance in Zone - Zone 16 | Distance in Zone (%) - Zone 16 | Time in Zone (Seconds) - Zone 16 | Time in Zone (%) - Zone 16 | Total Distance (cm) | Total speed (cm/s) (240s) | Time in Zone (Seconds) - Open Arms | Time in Zone (%) - Open Arms | Distance in Zone - Open Arms | Distance in Zone (%) - Open Arms | Total Distance | Total speed (cm/s) (240s) |
| Control1 | 251.76 | 19.52 | 55.63 | 23.18 | 1289.73 | 5.37 | 7.30 | 3.04 | 128.49 | 5.23 | 2456.72 | 10.24 |
| Control2 | 440.55 | 27.78 | 94.63 | 39.43 | 1585.87 | 6.61 | 7.49 | 3.12 | 109.74 | 4.11 | 2670.14 | 11.13 |
| Control3 | 273.47 | 26.75 | 71.38 | 29.74 | 1022.30 | 4.26 | 7.18 | 2.99 | 130.26 | 3.67 | 3549.36 | 14.79 |
| Control4 | 167.35 | 15.96 | 54.38 | 22.66 | 1048.54 | 4.37 | 7.01 | 2.92 | 68.43 | 2.98 | 2296.35 | 9.57 |
| Control5 | 238.61 | 17.76 | 43.75 | 18.23 | 1343.50 | 5.60 | 9.55 | 3.98 | 136.97 | 4.11 | 3332.61 | 13.89 |
| Control6 | 248.49 | 21.55 | 60.43 | 25.18 | 1153.09 | 4.80 | 7.92 | 3.30 | 117.34 | 4.65 | 2523.41 | 10.51 |
| Control7 | 181.34 | 16.30 | 62.45 | 26.02 | 1112.52 | 4.64 | 6.38 | 2.66 | 54.19 | 2.51 | 2158.99 | 9.00 |
| Control8 | 216.26 | 20.60 | 75.43 | 31.43 | 1049.80 | 4.37 | 5.64 | 2.35 | 55.89 | 2.73 | 2047.35 | 8.53 |
| Model1 | 116.94 | 14.19 | 27.62 | 11.51 | 824.10 | 3.43 | 5.35 | 2.23 | 32.35 | 2.53 | 1278.79 | 5.33 |
| Model2 | 152.90 | 15.26 | 33.74 | 14.06 | 1001.96 | 4.17 | 4.32 | 1.80 | 30.08 | 1.97 | 1526.88 | 6.36 |
| Model3 | 129.30 | 14.65 | 30.07 | 12.53 | 882.57 | 3.68 | 4.06 | 1.69 | 27.49 | 1.81 | 1518.72 | 6.33 |
| Model4 | 110.75 | 13.18 | 23.69 | 9.87 | 840.26 | 3.50 | 4.01 | 1.67 | 25.54 | 1.92 | 1329.96 | 5.54 |
| Model5 | 116.19 | 14.32 | 37.13 | 15.47 | 811.39 | 3.38 | 3.70 | 1.54 | 17.70 | 1.78 | 994.58 | 4.14 |
| Model6 | 114.34 | 13.22 | 34.25 | 14.27 | 864.91 | 3.60 | 5.04 | 2.10 | 24.36 | 1.92 | 1268.96 | 5.29 |
| Ven1 | 220.27 | 21.73 | 61.68 | 25.70 | 1013.67 | 4.22 | 5.62 | 2.34 | 74.93 | 2.61 | 2870.85 | 11.96 |
| Ven2 | 294.05 | 25.49 | 72.43 | 30.18 | 1153.60 | 4.81 | 5.81 | 2.42 | 72.69 | 2.81 | 2586.98 | 10.78 |
| Ven3 | 252.69 | 20.59 | 56.45 | 23.52 | 1227.26 | 5.11 | 8.76 | 3.65 | 70.54 | 3.35 | 2105.66 | 8.77 |
| Ven4 | 177.80 | 19.11 | 74.38 | 30.99 | 930.38 | 3.88 | 6.26 | 2.61 | 72.47 | 2.80 | 2588.16 | 10.78 |
| Ven5 | 201.56 | 18.40 | 94.01 | 39.17 | 1095.44 | 4.56 | 6.74 | 2.81 | 53.97 | 2.75 | 1962.49 | 8.18 |
| Ven6 | 234.71 | 19.67 | 48.86 | 20.36 | 1193.25 | 4.97 | 8.50 | 3.54 | 73.26 | 3.60 | 2035.04 | 8.48 |
| ZZCD1 | 177.88 | 18.69 | 81.62 | 34.01 | 951.73 | 3.97 | 7.15 | 2.98 | 59.73 | 3.02 | 1977.81 | 8.24 |
| ZZCD2 | 305.11 | 23.10 | 87.94 | 36.64 | 1320.83 | 5.50 | 7.32 | 3.05 | 80.16 | 2.99 | 2681.02 | 11.17 |
| ZZCD3 | 238.17 | 21.68 | 55.25 | 23.02 | 1098.55 | 4.58 | 7.37 | 3.07 | 59.79 | 3.10 | 1928.69 | 8.04 |
| ZZCD4 | 243.03 | 21.16 | 80.38 | 33.49 | 1148.52 | 4.79 | 8.57 | 3.57 | 172.16 | 6.76 | 2546.68 | 10.61 |
| ZZCD5 | 214.49 | 19.50 | 59.50 | 24.79 | 1099.94 | 4.58 | 6.00 | 2.50 | 70.05 | 2.81 | 2492.73 | 10.39 |
| ZZCD6 | 205.70 | 16.83 | 46.32 | 19.30 | 1222.24 | 5.09 | 6.12 | 2.55 | 67.35 | 2.78 | 2422.76 | 10.09 |
| ZZCD7 | 236.93 | 17.10 | 73.56 | 30.65 | 1385.56 | 5.77 | 6.31 | 2.63 | 54.57 | 2.50 | 2182.93 | 9.10 |

**Table S6. DEGs of NAc tissues from model mice after ZZCD administration.**

| Gene Symbol | Gene ID | Model1A FPKM | Model2A FPKM | Model3A FPKM | ZZCD1A FPKM | ZZCD2A FPKM | ZZCD3A FPKM |
| --- | --- | --- | --- | --- | --- | --- | --- |
| Ap1m2 | 11768 | 0.4 | 0.19 | 0.21 | 0.42 | 0.34 | 0.6 |
| Arc | 11838 | 22.32 | 21.07 | 35.05 | 53.38 | 55.15 | 87.93 |
| Bcl3 | 12051 | 0.41 | 0.18 | 0.67 | 0.1 | 0.17 | 0.24 |
| Bdh2 | 69772 | 1.16 | 1.19 | 0.97 | 0.17 | 0.91 | 0.58 |
| Btg2 | 12227 | 3.36 | 3.03 | 5.01 | 9.1 | 8.05 | 10.01 |
| Casp4 | 12363 | 0.17 | 0.23 | 0.79 | 0.17 | 0.25 | 0.05 |
| Casr | 12374 | 0.08 | 0.09 | 0.05 | 0.03 | 0.06 | 0.08 |
| Cd36 | 12491 | 0.05 | 0.02 | 0.09 | 0.19 | 0.2 | 0.22 |
| Ch25h | 12642 | 0.32 | 0.38 | 3.06 | 1.11 | 0.05 | 0.54 |
| Cntf | 12803 | 0.37 | 0.49 | 0.42 | 2.62 | 0.73 | 0.66 |
| Col4a3 | 12828 | 0.04 | 0.08 | 0.04 | 0.07 | 0.09 | 0.1 |
| Cyp2a5 | 13087 | 0.33 | 0.83 | 0.32 | 0.05 | 0.18 | 0.21 |
| Cyp2e1 | 13106 | 0.19 | 0.05 | 0.1 | 0.17 | 0.21 | 0.13 |
| Cysltr2 | 70086 | 0.08 | 0.11 | 0.23 | 0.15 | 0.21 | 0.26 |
| Dapk2 | 13143 | 0.11 | 0.25 | 0.24 | 0.47 | 0.14 | 0.58 |
| Dusp1 | 19252 | 10.42 | 11.04 | 15.43 | 31.92 | 21.29 | 30.79 |
| Egr2 | 13654 | 1.73 | 1.42 | 1.67 | 3.1 | 4.18 | 4.22 |
| Epha2 | 13836 | 0.27 | 0.36 | 0.42 | 1.03 | 0.51 | 0.41 |
| F2rl1 | 14063 | 0.02 | 0.05 | 0.04 | 0.09 | 0.03 | 0.08 |
| Fcgr4 | 246256 | 0.2 | 0.16 | 0.75 | 0.29 | 0.1 | 0.2 |
| Fos | 14281 | 5.54 | 3.25 | 5.14 | 14.03 | 15.93 | 18.89 |
| Galr3 | 14429 | 0.1 | 0.11 | 0.05 | 0.15 | 0.15 | 0.05 |
| Gast | 14459 | 0.74 | 0.46 | 0.88 | 0.14 | 0.29 | 0.14 |
| Glra1 | 14654 | 0.23 | 0.21 | 0.26 | 0.05 | 0.15 | 0.13 |
| Gm4767 | 210583 | 1.68 | 0.78 | 0.37 | 0.79 | 1.01 | 0.02 |
| Gm9839 | 408192 | 1.12 | 0.88 | 0.93 | 0.19 | 0.54 | 0.47 |
| Gnmt | 14711 | 0.35 | 0.36 | 0.43 | 0.73 | 0.61 | 0.96 |
| Guca1b | 107477 | 0.04 | 0.23 | 0.49 | 0.18 | 0.45 | 0.34 |
| Hpse2 | 545291 | 0.11 | 0.18 | 0.14 | 0.09 | 0.05 | 0.02 |
| Hspa1a | 193740 | 1.85 | 1.89 | 4.51 | 5.84 | 3.68 | 3.99 |
| Il1a | 16175 | 0.41 | 0.58 | 0.87 | 0.33 | 0.34 | 0.36 |
| Il27ra | 50931 | 0.36 | 0.11 | 0.16 | 0.13 | 0.11 | 0.19 |
| Il31ra | 218624 | 0.03 | 0.02 | 0.02 | 0.03 | 0.05 | 0.03 |
| Junb | 16477 | 33.47 | 34.07 | 38.67 | 61.76 | 64.47 | 62.36 |
| Kcnj15 | 16516 | 0.01 | 0.08 | 0.04 | 0.04 | 0.07 | 0.09 |
| Klf4 | 16600 | 1.8 | 1.78 | 2.21 | 3.31 | 2.66 | 3.49 |
| Krt17 | 16667 | 0.05 | 0.21 | 0.05 | 0.2 | 0.1 | 0.05 |
| Lcn2 | 16819 | 0.15 | 0.31 | 52.79 | 10.8 | 0.45 | 0.29 |
| Lhb | 16866 | 0.4 | 1.06 | 0.46 | 0.19 | 0.34 | 0.08 |
| Nr4a1 | 15370 | 32.01 | 25.24 | 27.72 | 44.03 | 58.58 | 68.16 |
| P2ry2 | 18442 | 0.11 | 0.08 | 0.17 | 0.11 | 0.24 | 0.3 |
| Slc24a1 | 214111 | 0.09 | 0.05 | 0.05 | 0.13 | 0.16 | 0.13 |
| Ssxb5 | 387586 | 0.65 | 0.08 | 0.7 | 0.15 | 0.24 | 0.28 |
| Stx11 | 74732 | 0.23 | 0.16 | 0.17 | 0.06 | 0.1 | 0.09 |
| Thbs1 | 21825 | 0.14 | 0.11 | 0.27 | 0.34 | 0.22 | 0.33 |
| Trim5 | 667823 | 0.6 | 0.68 | 0.66 | 0.26 | 0.57 | 0.51 |
| Ttr | 22139 | 0.08 | 0.5 | 0.24 | 0.46 | 0.87 | 0.63 |
| Twist1 | 22160 | 0.15 | 0.04 | 0.08 | 0.33 | 0.11 | 0.64 |

**Table S7. Binding energy of mRNA to material components of ZZCD**

|  | UniProt ID | SB236057A | Oleanolic acid |
| --- | --- | --- | --- |
| Egr2 | A0A7L3MEX5 | -7.18 | -6.36 |
| Dusp1 | A0A7L4AXQ0 | -8.22 | -7.44 |
| Fos | A0A7K4PAN9 | -6.46 | -5.58 |
| Junb | A0A6P8PFW5 | -6.37 | -6.46 |
| Nr4a1 | A0A383ZWZ0 | -8.01 | -7.36 |

**Table S8. Gastric emptying rates (GER) and small intestinal transit rates (ITR)**

|  | Con1 | Con2 | Con3 | Mod1 | Mod2 | Mod3 | Ven1 | Ven2 | Ven3 | ZZCD1 | ZZCD2 | ZZCD3 |
| --- | --- | --- | --- | --- | --- | --- | --- | --- | --- | --- | --- | --- |
| GER | 0.3329 | 0.1369 | 0.1551 | 0.0452 | 0.0713 | 0.0863 | 0.0998 | 0.0867 | 0.1094 | 0.1536 | 0.1855 | 0.1069 |
| ITR | 1 | 1 | 1 | 0.8974 | 0.8857 | 0.8378 | 1 | 0.95 | 0.923 | 1 | 0.9218 | 1 |
